# Bacterial communities in a subarctic stream network: Spatial and seasonal patterns of benthic biofilm and bacterioplankton

**DOI:** 10.1111/mec.16711

**Published:** 2022-10-17

**Authors:** Jacqueline Malazarte, Timo Muotka, Jussi Jyväsjärvi, Kaisa Lehosmaa, Joel Nyberg, Kaisa‐Leena Huttunen

**Affiliations:** ^1^ Ecology and Genetics Research Unit University of Oulu Oulu Finland

**Keywords:** amplicon sequence variant, bacteria, microbial diversity, river network, temporal

## Abstract

Water‐column bacterial communities are assembled by different mechanisms at different stream network positions, with headwater communities being controlled by mass effects (advection of bacteria from terrestrial soils) while downstream communities are mainly driven by environmental sorting. Conversely, benthic biofilms are colonized largely by the same set of taxa across the entire network. However, direct comparisons of biofilm and bacterioplankton communities along whole stream networks are rare. We used 16S rRNA gene amplicon sequencing to explore the spatiotemporal variability of benthic biofilm (2 weeks old vs. mature biofilm) and water‐column communities at different network positions of a subarctic stream from early summer to late autumn. Amplicon sequence variant (ASV) richness of mature biofilm was about 2.5 times higher than that of early biofilm, yet the pattern of seasonality was the same, with the highest richness in midsummer. Biofilm bacterial richness was unrelated to network position whereas bacterioplankton diversity was negatively related to water residence time and distance from the source. This pattern of decreasing diversity along the network was strongest around midsummer and diminished greatly as water level increased towards autumn. Biofilm communities were phylogenetically clustered at all network positions while bacterioplankton assemblages were phylogenetically clustered only at the most downstream site. Both early and mature biofilm communities already differed significantly between upstream (1st order) and midstream (2nd order) sections. Network position was also related to variation in bacterioplankton communities, with upstream sites harbouring substantially more unique taxa (44% of all upstream taxa) than midstream (20%) or downstream (8%) sites. Some of the taxa that were dominant in downstream sections were already present in the upmost headwaters, and even in riparian soils, where they were very rare (relative abundance <0.01%). These patterns in species diversity and taxonomic and phylogenetic community composition of the riverine bacterial metacommunity were particularly strong for water‐column communities, whereas both early and mature biofilm exhibited weaker spatial patterns. Our study demonstrated the benefits of studying bacterioplankton and biofilm communities simultaneously to allow testing of ecological hypotheses about biodiversity patterns in freshwater bacteria.

## 1 INTRODUCTION

Bacterial communities in streams are assembled by a continuum of mechanisms along the riverine network, with headwater communities being controlled by the advection of bacteria from terrestrial soils (mass effects) while those in downstream sections are mainly driven by strong environmental selection (species sorting) (Crump et al., 2012; Hassell et al., 2018; Nelson et al., 2009; Niño‐García et al., 2016a; Ruiz‐González et al., 2015). The land–water linkage is strongest at the upmost headwaters which receive high rates of soil‐derived immigrants, particularly during rainfall‐induced flood events (Caillon et al., 2021; Teachey et al., 2019). As water residence time (WRT) in headwaters is short, their bacterioplankton community composition probably reflects environmental sorting in the source habitat (e.g., soil pH or moisture) (Niño‐García et al., 2016a). Lower down in the network, aquatic habitat filters predominate, and the numbers of soil‐derived taxa, although still comprising a large proportion of the community, are greatly reduced in downstream sections (Ruiz‐González et al., 2015; Savio et al., 2015). Consequently, the taxonomic diversity of the water‐column bacterial community typically decreases along the flow path and, although much less studied, a corresponding trend has also been observed for benthic biofilm communities (Besemer et al., 2013). Some bacterial taxa that are very rare in upstream soils are well adapted to the freshwater environment and can become abundant or even dominant at larger river sites (“core seed bank”; Ruiz‐Gonzalez et al., 2017a). In addition, Read et al. (2015) found that bacterial activity rates were higher in the upstream tributaries than in downstream sections of the River Thames, UK, indicating that many of the taxa derived from terrestrial sources remain dormant in headwaters. Thus, community assembly mechanisms are modulated by the position of a site within the network, with soil taxa being a part of the riverine metacommunity as a source for downstream communities (Crump et al., 2012; Hermans et al., 2020; Nino‐Garcia et al., 2016b),

The relative importance of environmental selection also differs between different in‐stream habitats, and the two key components of the riverine microbiome, bacterioplankton and benthic biofilm, are structured differently over space and time. While environmental filters select for similar bacterioplankton communities only in downstream sections, biofilm is colonized by a largely similar set of taxa across the whole network (Wisnoski & Lennon, 2021). For example, Besemer et al. (2012) noticed that while bacterioplankton communities varied substantially among sites, biofilm communities did not; consequently, they concluded that stochastic dispersal from bacterioplankton did not regulate biofilm community assembly. Rather, biofilm consists of a core set of taxa with traits beneficial in benthic environments, and different stream habitats thus support largely distinct microbial lifestyles (sensu Ezzat et al., 2022; Niederdorfer et al., 2016). As a result of strong environmental filtering in benthic habitats, biofilm bacterial communities are usually less diverse than those in the overlying water column (Besemer et al., 2012; Niederdorfer et al., 2016; but see Gweon et al., 2021). Biofilm community assembly could be expected to be less dependent on the advection of soil‐derived taxa compared to bacterioplankton and they should therefore exhibit a weaker, if any, diversity pattern along the stream network (but see Besemer et al., 2013). Network position may thus have different implications for the water‐column and biofilm communities: for bacterioplankton, position relates to water residence time and distance from the source community whereas for biofilm, network position mainly indicates that the overlying bacterial species pool is different. Therefore, the location of a site within the network could be more critical for bacterioplankton than biofilm communities.

Biofilm development involves a series of successional stages, with corresponding differences in community composition and diversity (Jackson, 2003). Early developmental stages are largely stochastic, reflecting colonization from the water column (Lyautey et al., 2005; Woodcock & Sloan, 2017); thus, species richness initially increases rapidly, then reaches a plateau, or even decreses slightly (Jackson, 2003). Through time, the architectural complexity of biofilm increases and more specialized taxa begin to establish; as a result, species richness may increase again (Battin et al., 2016). These later stages of biofilm formation are controlled by deterministic factors, leading bacterial communities in mature biofilm to diverge from the more stochastic early stages (Veach et al., 2016). This successional pattern is often paralleled by a major shift in biofilm functioning. The initial colonizing populations are heterotrophic, being dependent on the dissolved organic carbon (DOC) in the stream water, while the later stages are net autotrophic (Jackson, 2003; Veach et al., 2016).

Temporal variability of freshwater bacterial communities has been much less studied than its spatial counterpart although temporal patterns in community composition can be even stronger than those occurring through space (Portillo et al., 2012; Veach et al., 2016). Some evidence, mainly from lentic environments, suggests that temporal patterns in bacterial communities may be relatively predictable (Shade et al., 2013; Veach et al., 2016), with annually recurring seasonal variability in community composition (Crump and Hobbie, 2005; David et al., 2021; Staley et al., 2015). Water temperature (Hullar et al., 2006; Teachey et al., 2019) and river discharge (Fortunato et al., 2012) are the key environmental factors reported to underlie seasonal variability in bacterial communities. Thus, in seasonally predictable environments, lotic communities tend to be highly stable at interannual scales but, at the same time, exhibit rapid turnover on weekly or even daily scales following abrupt environmental shifts in stream conditions (Caillon et al., 2021; Portillo et al., 2012; Veach et al., 2016).

We explored the spatiotemporal variability of bacterial communities in a subarctic stream network in northeastern Finland. We sampled 13 sites at different network positions from the upmost 1st‐order headwaters to 3rd‐order river reaches. The sites were sampled repeatedly through time in 2‐ to 3‐week intervals to capture the full range of open‐water seasonal variability (early June to October) in water temperature, stream discharge and water chemistry. Our sampling covered both benthic biofilm and water‐column bacterial communities. We hypothesized (i) bacterioplankton diversity to exhibit a longitudinally decreasing trend which should be disrupted in autumn as water level (and thus water residence time) increases as a result of increased precipitation; the corresponding pattern for biofilm communities should be weaker or non‐existent. Furthermore, we expected (ii) bacterioplankton communities to vary more along the river network than biofilm communities, because the latter should respond to a largely similar set of habitat filters at all network positions. Next, we expected (iii) different stages of biofilm formation (early vs. mature) to differ in bacterial community composition, with mature biofilm being characterized by the presence of autotrophic bacteria. Reflecting the strong environmental filtering in benthic habitats, we expected (iv) biofilm communities to be phylogenetically more clustered than bacterioplankton. Finally, we expected that (v) bacterioplankton should exhibit a downstream shift in phylogenetic community structure, with upstream sites consisting of a phylogenetically variable array of both freshwater and soil‐derived taxa; these terrestrial taxa are mainly poorly adapted to flowing water and are therefore rapidly filtered out from the regional (metacommunity) species pool.

## 2 MATERIAL AND METHODS

### 2.1 Study system

**FIGURE 1:**
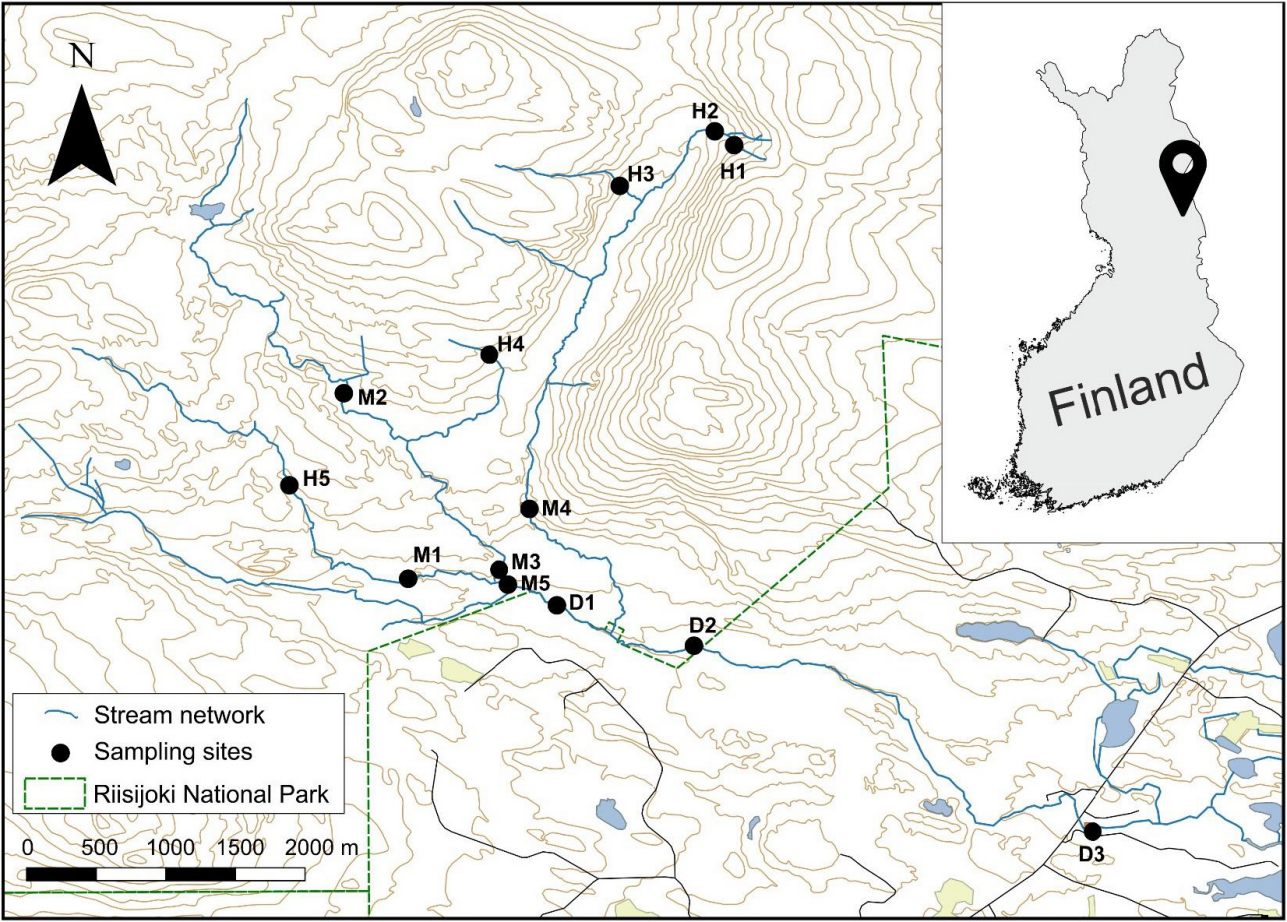
Location of study sites in the Riisijoki river network in northeastern Finland.

Our study system, River Riisijoki, has a catchment area of 27.6 km^2^ most of which is located within the Riisitunturi National Park in northeastern Finland (Figure 1). The river runs through coniferous forests and extensive peatlands before draining into Lake Kitkajärvi. The river and its catchment are in a pristine condition, except for the downmost 3‐km section of the main channel which is under commercial forestry and sparse settlement. Mean air temperature in the area is ~0°C and mean temperature is 15°C during the warmest month (July) and −12°C during the coldest month (January). Mean annual precipitation is 600–650 mm, 40% of which falls as snow. The stream remains ice‐covered for about 6 months, from early November to early May. Mean annual discharge is 0.34 m^3^ s^−1^, with a snowmelt‐induced peak of ~5 m^3^ s^−1^ in late May. Stream water is slightly acidic (pH 5.4–6.7), oligo‐ to mesotrophic (tot‐P: 8–25 μg L^−1^; tot‐N: 10–320 μg L^−1^) and brown‐coloured (DOC: 8–17 mg L^−1^).

We sampled 13 sites, representing the stream network from the upmost headwaters to the downmost site close to the lake inlet (see Figure 1). Headwater sites (hereafter: upstream) are first‐order streams (<1 m wide, *n* = 5), midstream sites are second‐order streams (2–4 m, *n* = 5) and downstream sites represent third‐order streams (6–11 m, *n* = 3). The upmost headwater site has a dendritic distance of 238 m from the source while the lowest site is located at 10,047 m away from the source. Sampling was carried out during the open‐water season (early June to early October) in 2‐ to 3‐week intervals; during the winter months, most of the river is inaccessible due to extensive snow and ice cover.

### 2.2 Sampling protocol

We sampled benthic biofilm both from colonization tiles (“early biofilm”) and natural stones (“mature biofilm”). For early biofilm, unglazed ceramic tiles (10 × 10 cm; *n* = 4 per site) were used as substrate and were left on site for 2–3 weeks to allow biofilm colonization. After incubation, a pooled biofilm sample was collected by wiping each of the four tiles with a sterile Speci‐Sponge (Whirl‐Pak, Nasco). On each sampling occasion, a new set of sterile tiles was placed on site to initiate new biofilm colonization. Mature biofilm was sampled simultaneously by randomly selecting four cobble‐sized natural stones and wiping the upper biofilm surface using a Speci‐Sponge. Biofilm samples were placed in a sterile 50‐ml centrifuge tube in the field and stored at −20°C upon arrival at the laboratory. For bacterioplankton, three replicate water samples were collected into sterile 50‐ml centrifuge tubes along a transect perpendicular to the stream flow and the replicates were pooled prior to molecular analyses.

To test whether the bacterial taxa that dominated in downstream sections originated from upstream soils, we sampled riparian soil communities parallel to other biological sampling at each upstream site. Soil samples were collected at ~1‐m distance from the stream edge to a depth of 10 cm using a 2.5‐cm‐diameter soil corer. Four replicate samples were collected and pooled to obtain a composite sample for each site. Samples were stored at −20°C until further processing.

In parallel with biological sampling, we collected water samples at each site into 500‐ml plastic bottles and analysed them for pH, DOC (mg L^−1^), total phosphorus (TP, μg L^−1^) and nitrate‐nitrite nitrogen (NO^3^‐NO^2^‐N, μg L^−1^) using Finnish national standards (National Board of Waters and the Environment, 1981). Groundwater contribution at each site was estimated as the ratio of ^18^O to ^16^O stable isotopes (δ^18^O) of the stream water (see Lehosmaa et al., 2017) using cavity ringdown spectroscopy with a Picarro L2120‐i analyser (Picarro). Filtered 50‐ml subsamples (0.7 μm, Whatman GF/F) were measured for absorbance at 436 nm using a Shimadzu UV‐160A spectrophotometer. Absorbance was used as a measure of the amount of coloured dissolved organic matter (DOM) in the total DOM (Ask et al., 2009).

We also quantified algal biomass accrual rate by using similar colonization tiles as for biofilm (5 × 5 cm, *n* = 4 replicate tiles per site). The tiles were changed at 2‐ to 3‐week intervals and periphyton accumulated on the upper tile surfaces was scraped off with a toothbrush. The algal samples were analysed for chlorophyl‐a (chl‐a) content in the laboratory by acetone extraction with subsequent spectrophotometric fluorescence readings. We averaged replicate measurements to give a single value for algal biomass accumulation (μg cm^−2^ day^−1^) at each site and time. Water velocity was measured 1–2 cm above each tile on each visit to a site. We also measured stream water temperature and water level at 30‐min intervals from late May to early October at each site using data loggers (WT‐HR 1000 mm, TruTrack Ltd). In subsequent data analyses, we used mean values across the 7‐day period preceding each bacterial sample for water temperature; for water level, we used daily means for the 7‐day period prior to sampling.

### 2.3 Molecular analyses and bioinformatics

Microbial DNA was extracted from water samples (bacterioplankton), sponges (biofilm) and soil samples (25 g). Prior to filtration, 100 ml of molecular‐grade water was added to the sponges and bacterial biofilms were extracted using a Stomacher 400 Circulator at 260 r.p.m. for 2 min. Water and biofilm samples were filtered with sterilized 0.2‐μm nylon membrane (Whatman) filters and DNA for all three sample types were extracted with Qiagen's DNeasy PowerSoil DNA Isolation kit following the manufacturer's recommendations. The V4–V5 region of the 16S rRNA gene was amplified using 519F and R926 primers (Quince et al., 2011). Triplicate 20‐μl polymerase chain reactions (PCRs) contained 10 ng of template DNA, 1× Phusion GC buffer, 0.5 μm of forward and reverse primers, 0.2 mm dNTPs and 0.4 U of Phusion high‐fidelity DNA polymerase (S7 Fusion Polymerase, MOBIDIAG). The amplification consisted of an initial denaturation step at 98°C for 30 s, 30 cycles of denaturation at 98°C for 10 s, annealing at 64°C for 30 s, extension at 72°C for 20 s and a final extension step at 72°C for 5 min. The PCR amplicon triplicates were pooled (sample volume after pooling = 60 μl) after which pooled samples were purified with AmpureXP PCR purification reagent (Agencourt Bioscience) and quantified using Bioanalyzer DNA‐1000 chips (Agilent Technologies). Prior to sequencing, each sample was quantified with the PicoGreen dsDNA assay kit (ThermoFisher) and, based on the quantity, diluted to an appropriate concentration. Ion Torrent sequencing was applied using an Ion Torrent Hi‐Q OT2 kit, Ion Torrent Hi‐Q View Sequencing kit and 316 v2 chip with sequencing length of 400 bp (ThermoFisher).

Sequences were processed using the qiime2 (version 2020–8) microbiome bioinformatics pipeline (Bolyen et al., 2019). Short reads (<100 bp) were removed before remaining reads were demultiplexed with sample‐specific barcodes using the *q2‐cutadapt* plugin (Martin, 2011). Single‐end demultiplexed sequences were further processed with dada2 using the *denoise‐pyro* option with truncate length of 220 nucleotides (Callahan et al., 2016). After denoising, four separate Ion Torrent sequencing runs were combined as one qiime2 artefact. Amplicon sequence variants (ASVs; Callahan et al., 2017) were aligned to the silva 16S version 138.1 Gene Database (Quast et al., 2013) with primer trimmed (mean read count of 8778, range 2244–68,895) data using the pretrained classify‐sklearn naïve Bayes taxonomy classifier (via the *q2‐feature‐classifier*; Bokulich et al., 2018). Pretraining of the classifier was done using reference reads that were first modified to qiime2 format and then extracted using the primers used in the amplification. Prior to further analyses, singletons, mitochondria, chloroplasts and unassigned sequences were removed. Aligned sequences of ASVs were used to construct a maximum‐likelihood tree in fast‐tree (Price et al., 2010) to include the phylogeny of bacteria in further analyses.

### 2.4 Statistical analyses

We divided the sites into three groups relative to network position, based on stream order and dendritic network distance (i.e., length [m] of the river network upstream of a sampling site) (see Figure 1). We measured patterns in α‐diversity, separately for bacterioplankton and benthic biofilm communities, using Hill numbers of order *q* (^*q*^D), with *q* = 0 (species richness), 1 (Shannon entropy) and 2 (inverse of Simpson index). The value of ^*q*^D is the number of equally abundant species needed to obtain the observed value of a diversity metric (“effective species number”). Thus, increasing the value of *q* progressively weighs down the influence of rare species on the index value (e.g., Chao et al., 2014).

We used two‐way repeated‐measures analysis of variance (ANOVA) on rarefied (down to 1140 sequences; the lowest shared sequence number) ASV richness to test for differences in network position, sampling time and their interaction on taxonomic diversity (Hill numbers). For statistical analyses, we simplified the temporal aspect of our study design by pooling samples into three time periods (seasons): early summer (from late May to early July), midsummer (early July to mid‐August) and autumn (mid‐August to early October). Pairwise differences were examined with Tukey's test. We also related Hill numbers to WRT, in a simple linear regression. WRT, being a continuous measure describing stream network positions, was calculated based on the measurement of water velocity at each visit to a site and the total upstream distance (see Niño‐García et al., 2016a).

Variation in bacterial community composition among network positions (upstream, midstream, downstream) and seasons (early summer, midsummer, autumn) was normalized using the total sum scaling (TSS) method and visualized with nonmetric multidimensional scaling (NMDS). Significance among groups was tested using nonparametric permutational multivariate analysis of variance (PERMANOVA) with the *adonis* function in vegan (Oksanen et al., 2020). PERMANOVAs were run using Bray–Curtis similarities on relative abundance data and statistical significance was estimated based on 9999 permutations. The argument *strata* in the “*adonis2*” function was used to account for temporally replicated sampling at each site. A significant global test was followed by pairwise PERMANOVAs between different network positions. The function “*envfit*” of vegan was used to detect the direction in the ordination space towards which environmental vectors change most rapidly and have maximal correlation with the ordination configuration. The strongest predictors were then plotted onto the ordination diagram as arrows, with the length of the arrow indicating the relative importance of a predictor. The environmental variables included in envfit were water temperature, water depth (mean and coefficient of variation [CV]), water velocity, chl‐*a* concentration, pH, tot‐P, NO_3_ + NO_2_, DOC, groundwater contribution (δ^18^O) and absorbance_436_.

To characterize phylogenetic community composition, we calculated the “mean nearest taxon distance” (α‐MNTD_obs_), that is the mean phylogenetic distance to the closest relative for each ASV at each sample (Fine & Kembel, 2011; Stegen et al., 2012). To generate a null expectation for MNTD, the ASVs were randomly placed across the tips of the phylogeny (“taxa. labels” in function ses.mntd, R package picante 1.8.2; Kembel et al., 2010) and MNTD was recalculated to provide a null MNTD value (MNTD_null_). This procedure was repeated 999 times to provide a distribution of MNTD_null_ values to which MNTD_obs_ was compared. Next, we calculated the “nearest taxon index” (NTI) which quantifies, in units of standard deviation, the difference between MNTD_obs_ and mean MNTD_null_ ([Obs‐Exp]/*SD*_Exp_, with 999 randomizations). For a single sample, NTI < −2 indicates that taxa within the sample are phylogenetically clustered (more closely related than expected by chance) whereas NTI > +2 indicates phylogenetic overdispersion (taxa are more distantly related than expected by chance). We then regressed these community‐wide measures of average phylogenetic distance against WRT to test whether network position was related to phylogenetic community composition. For this analysis, we used site‐specific values averaged through time, whereas season‐specific averaged values were used to examine temporal variability in phylogenetic clustering in each community type.

We used the R package ancom‐bc (analysis of composition of microbiomes with bias correction; Lin & Peddada, 2020) to test which bacterial taxa exhibited the strongest spatial affinity (i.e., differed most in abundance across network positions). ancom‐bc is based on the linear regression framework and aims to identify differentially abundant taxa between categories while correcting the bias introduced by among‐sample differences in sampling fractions. It provides an improvement over previous techniques used for differential abundance testing by yielding a valid statistical test with adequate power and associated *p*‐values. It also provides confidence intervals for the differential abundance of each taxon and controls the false discovery rate in multiple testing (Lin & Peddada, 2020). We ran ancom‐bc separately for early and mature biofilm and bacterioplankton communities.

## 3 RESULTS

### 3.1 Environmental variables

**FIGURE 2:**
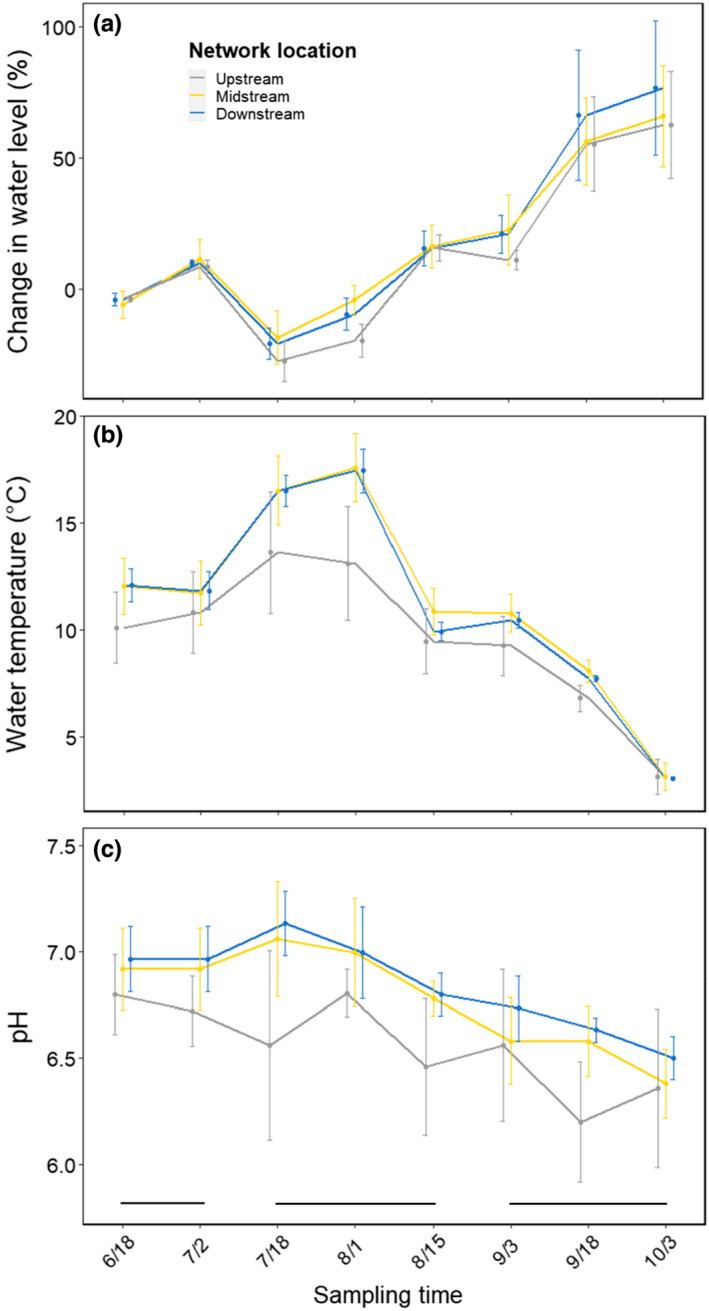
Mean (±1 SD) percentage change in water level (a), mean (± 1 SD) water temperature (b), averaged across a 7‐day period prior to each sample date, and pH (c) at different network positions through time. For (a), day 1 of the study is used as a reference. The horizontal bars indicate the seasons spanned by the study (early summer; midsummer; autumn).

Water level in our study streams was lowest in July, increasing gradually towards autumn (Figure 2a). This shift reflected the autumnal increase in precipitation (Figure S1a). Groundwater contribution exhibited a reversed pattern, with the lowest contribution (highest δ^18^O values) at all network positions in autumn (Figure S1b). Water temperature reached the highest values (around +15°C) in mid‐July and then decreased sharply to about +2°C in early October (Figure 2b). Water pH was lowest at upstream sites but with a similar seasonal pattern at all stream sections, with peak values in June to July and the lowest values (~6.5) in late autumn (Figure 2c).

### 3.2 Diversity patterns

**FIGURE 3:**
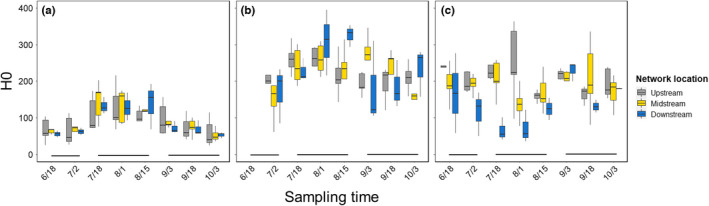
ASV richness (H0) across time at each of the three network positions (upstream; midstream; downstream) for early biofilm (a), mature biofilm (b) and bacterioplankton (c). Boxes represent median values (horizontal lines) with upper and lower quartiles; whiskers indicate the range of nonoutliers. The horizontal bars indicate the grouping of seasons (early summer; midsummer; autumn) for statistical analyses (for further detail, see text). One sample (early June) for mature biofilm was lost during laboratory processing.

Bacterial diversity displayed largely similar trends for all three measures (Hill numbers) and we therefore focus here mainly on ASV richness (other index values are displayed in Figure S2). The main effect of season on the early biofilm ASV richness was significant (*F*_2,20_ = 40.05, *p* < .001) whereas network position had no effect (*F*_2,10_ = 0.20, *p* = .82) (Figure 3a). The interaction term (position*season) was also insignificant (*F*_4,20_ = 0.40, *p* = .84). ASV richness was higher in midsummer than in either early summer or autumn samples (Tukey's test, both *p* < .001) (Figure 3a). ASV richness of mature biofilm was generally about 2.5 times higher than that of the early biofilm. Nevertheless, the pattern of seasonality was similar (*F*_2,20_ = 4.40, *p* = .026), with the highest ASV richness in midsummer samples and lower richness in both early summer (*p* = .015) and autumn (*p* = .089) (Figure 3b). Network location was clearly insignificant also for mature biofilm (*F*_2,10_ = 0.03, *p* = .97) as was also the interaction term (F_2,20_ = 0.61, *p* = .66).

For bacterioplankton, only network position had a significant main effect on ASV richness (F_2,10_ = 6.09, *p* = .019), with the highest diversity in the upmost sites, followed by much lower values in midstream and even lower in downstream sections (Figure 3c). This pattern was, however, significant only for the upstream vs. downstream comparison (Tukey's test, *p* = .015). Bacterioplankton displayed no seasonality in any of the diversity measures (all *p* > .20) (Figure 3c; Figure S2c,f) whereas the interaction term (season*position) was significant (*F*_4,20_ = 2.97, *p* = .045), reflecting the very low midsummer bacterioplankton richness in downstream sections compared to other network locations and disruption of the diversity gradient towards autumn (Figure 3c).

**FIGURE 4:**
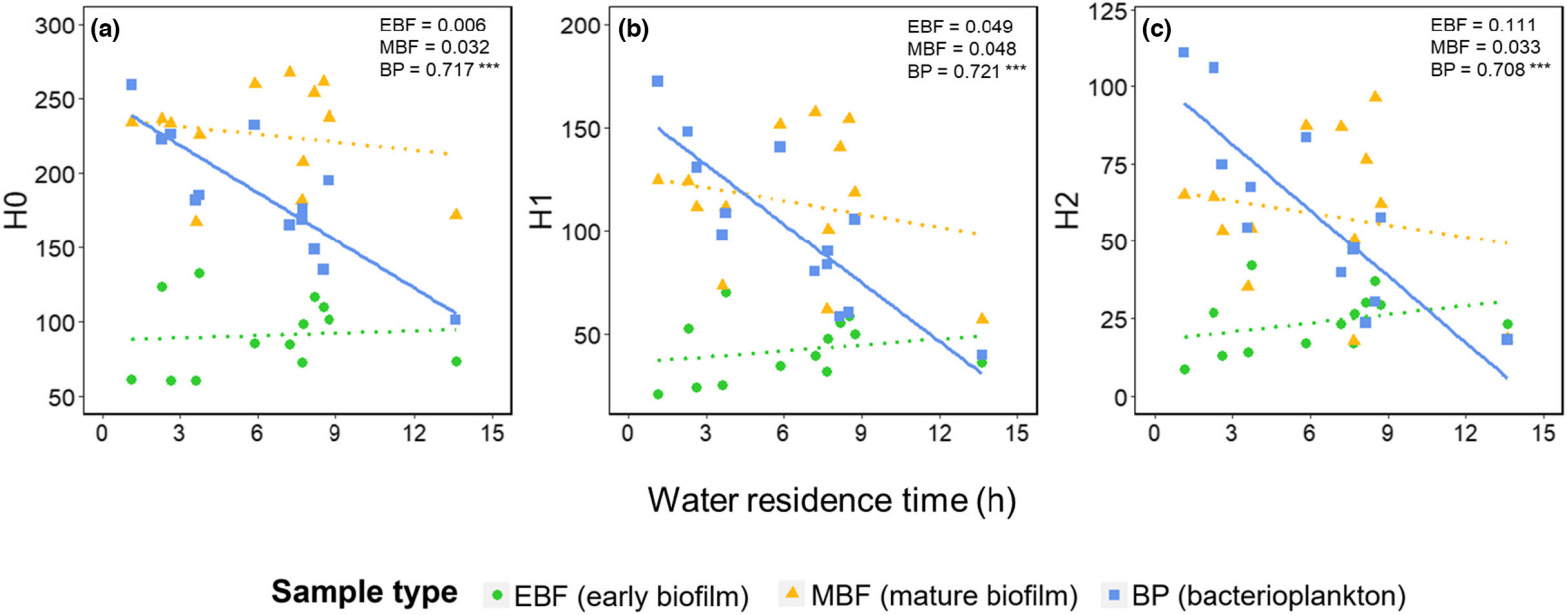
Relationship (linear regressions) of hill numbers with water residence time (h) along the river network: (a) ASV richness (H0), (b) Shannon diversity (H1) and (c) inverse Simpson index (H2). Significant patterns are displayed as solid lines. Data were averaged for each site across all sampling times. *R* ^2^ values are shown at the upper right corner with asterisks indicating significance: ***p < .001.

All three measures of diversity (Hill numbers 0–2, averaged across sampling times) were strongly negatively related to water residence time for bacterioplankton (Figure 4a–c). This pattern was particularly distinct for inverse Simpson index, with downstream sites being exceedingly dominated by a few key taxa (see also Figure S4; Tables S1–S3). No corresponding spatial pattern was detected for benthic biofilm.

### 3.3 Community composition

#### 3.3.1 Patterns of dominance

All community types were characterized by strong dominance but with a different pattern: while the degree of dominance (percentage cumulative relative abundance of the five most dominant taxa) was relatively constant across the network for biofilm (Tables S1 and S2), it almost tripled for bacterioplankton, from an average of 13% in upstream sites to 38% in downstream sites (Table S3). Several of the taxa that were dominant in downstream sites were already observed at upstream sites, and even in upstream soils, but always in much lower relative abundance. For early biofilm, the mean relative abundance of the 10 most abundant downstream taxa was 4.1% (2.0%–12.7%); eight of these were also detected at the upstream sites but at much lower abundance (0.5%; range 0%–2.9%). Two of these taxa were also found in upstream soils, with mean relative abundance of 0.015%. Corresponding numbers for mature biofilm were 2.5% (1.0%–6.8) in downstream, 0.6% (0.003%–2.9%, *n* = 10 taxa) in upstream water and 0.02% (0.002%–0.15%, *n* = 2 taxa) in upstream soil communities. For bacterioplankton, mean relative abundance of the 10 dominant ASVs was 4.4% (1.6%–1%9.9) in downstream sites, 1.04% (0.06%–3%.3, *n* = 10) in upstream sites and 0.05% (0.002%–0.26%, *n* = 7) in upstream soils. Overall, upstream bacterioplankton communities resembled soil communities more than did either mid‐ or downstream bacterioplankton (see Figure S6), although all pairwise differences were clearly significant (PERMANOVA: soil vs. upstream bacterioplankton, *F*_1,77_ = 25.02, *p* < .001; soil vs. midstream bacterioplankton, *F*_1,77_ = 33.15, *p* < .001; soil vs. downstream bacterioplankton, *F*_1,61_ = 30.00, *p* < .001).

#### 3.3.2 Spatial patterns: Biofilm

Mature and, more so, early biofilm was dominated by Proteobacteria, which comprised >80% of early and >50% of mature biofilm in all network positions (Figure S3a,b). Bacteroidota (mean relative abundance: 16%) was the only other phylum that reached a relative abundance of 10% in early biofilm while Cyanobacteria (25%) were also important in mature biofilm, as were also Bacteroidota (12%).

**FIGURE 5:**
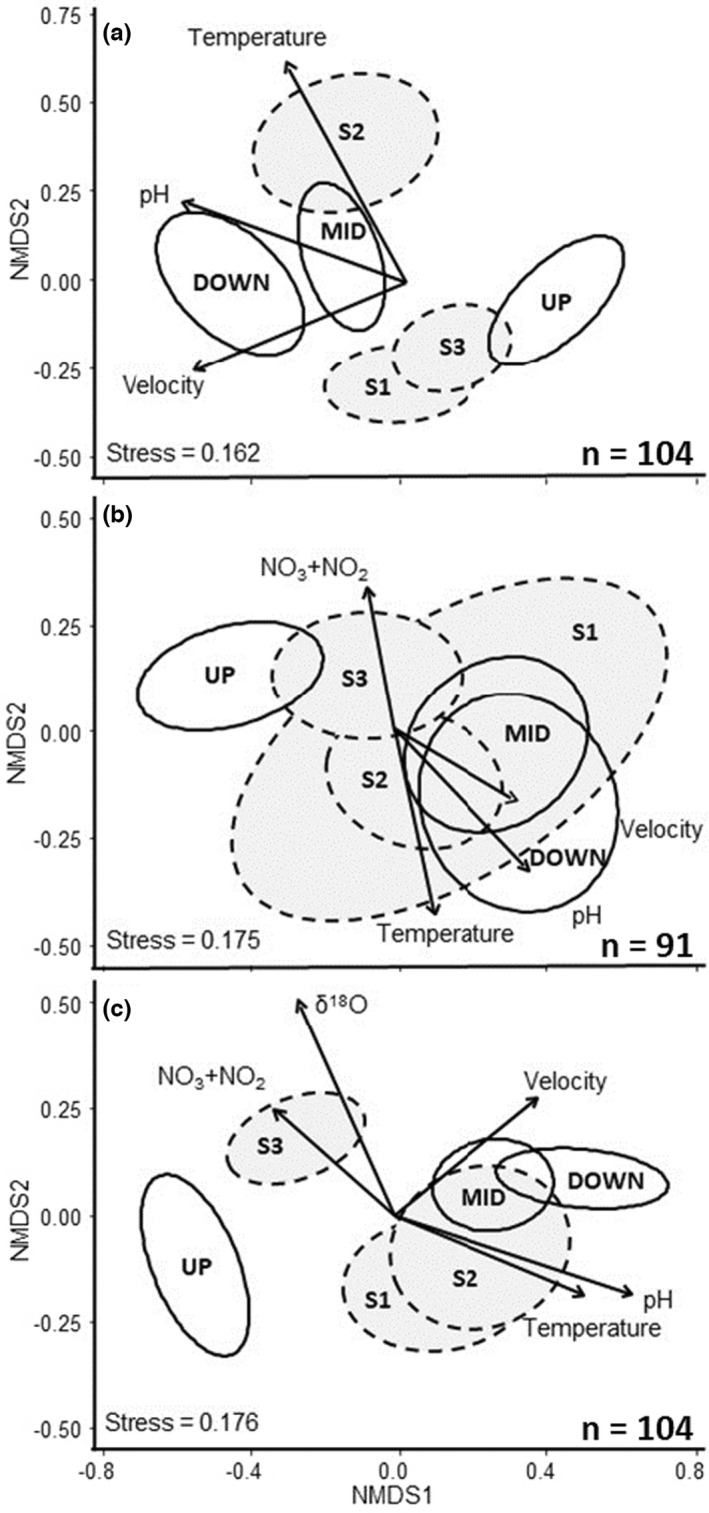
NMDS ordination of early (a) and mature (b) biofilm and bacterioplankton (c) communities based on ASV relative abundances at different study sites and seasons. The ellipses represent 95% confidence ellipses around group centroids. Open ellipses, continuous line: Spatial ordination; grey ellipses, broken line: Seasonal ordination. The strongest significant (at *p* < .01) environmental variables related to each ordination configuration are represented as arrows; length of an arrow is related to the relative importance of a variable. “*n*” represents the number of samples for each sample type.

**FIGURE 6:**
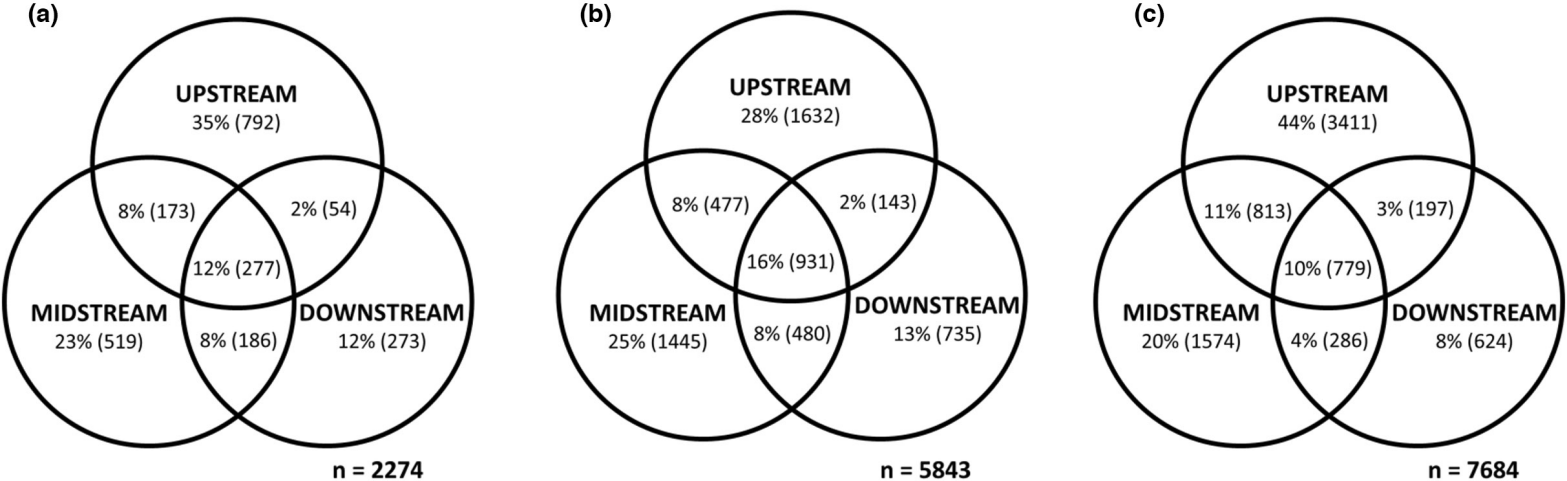
Venn diagrams displaying the numbers (and proportions) of taxa unique and shared between different network positions for early biofilm (a), mature biofilm (b) and bacterioplankton (c) communities. “*n*” represents the total number of ASVs for each sample type.

Network position contributed importantly to bacterial community composition in both early (PERMANOVA, global test: *F*_2,101_ = 6.02, *p* < .001) and mature biofilm (*F*_2,88_ = 4.46, *p* < .001). The most distinct community change occurred between the upstream and downstream sections for both early (*F*_1,62_ = 8.56, *p* < .001) (Figure 5a) and mature (*F*_1,54_ = 5.39, *p* < .001) (Figure 5b) biofilm although the difference between upstream and midstream communities was almost equally strong (early biofilm: *F*_1,78_ = 6.52, *p* < .001; mature biofilm: *F*_1,68_ = 6.14, *p* < .001). The abiotic variables that best correlated with the spatial community organization were the same for both biofilm types, namely pH and water velocity, both of which increased with increasing network distance (see Table S4). Upstream sites supported the most distinctive communities in terms of the proportion of unique taxa (taxa observed at only one network position) for both biofilm stages (Figure 6a,b). At the genus level, biofilm communities were dominated by a few very abundant taxa, particularly *Rhodoferax* (Gammaproteobacteria), that made up more than 65% of all genera in the early biofilm. *Rhodoferax* was still very abundant (up to 30%) in mature biofilm but was then replaced by the cyanobacterium *Scytonema* as the most dominant taxon (Figure S4a,b).

**FIGURE 7:**
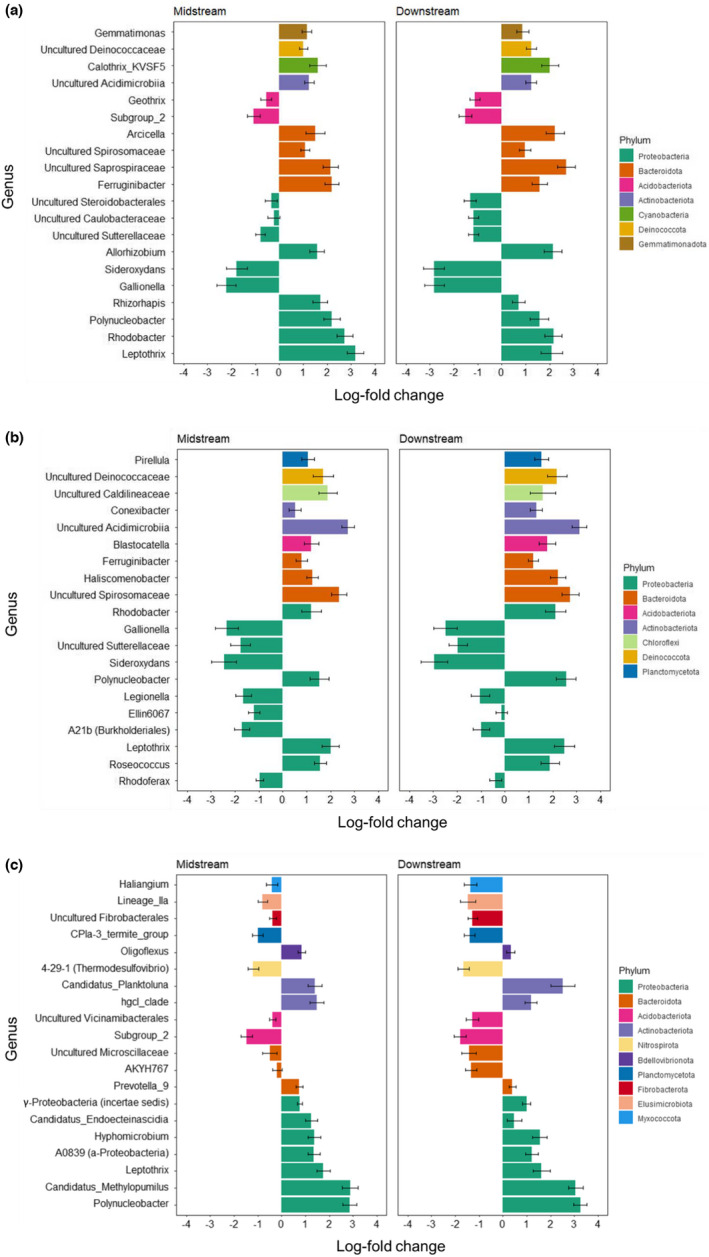
Bacterial genera identified by the analysis of composition of microbiomes with bias correction (ancom‐bc) as differentially abundant among different network positions: (a) early biofilm, (b) mature biofilm, (c) bacterioplankton. Bars represent the effect size (log‐fold change ±95% confidence intervals) for each genus, with negative values associated with significantly higher abundance in upstream sites and positive values with higher abundance in midstream (left panel) or downstream (right panel) sites.

Across all sampling times, 52 bacterial genera in early biofilm were identified by ancom‐bc as differentially abundant between upstream and downstream sites, and 40 genera between upstream and midstream sites; the majority of these were more abundant in mid‐ and downstream than in upstream sites (Figure 7a). Largely, the same taxa distinguished both midstream and downstream sites from the upstream sites, but they tended to differ more between upstream and downstream sites. For early biofilm, the two most distinctive upstream genera were *Sideroxydans* and *Gallionella* (both Gammaproteobacteria). Examples of genera that occurred at significantly higher abundance in midstream and/or downstream than upstream sites were an uncultured Saprospiraceae (Bacteroidia), *Rhodobacter* (class Alphaproteobacteria), *Leptothrix* (Gammaproteobacteria), *Ferruginibacter* (Bacteroidia) and *Calothrix* (Cyanobacteria) (Figure 7a).

For mature biofilm, 37 genera were significantly differentially abundant between upstream and midstream sites; corresponding numbers for the upstream–downstream comparison was 30. *Sideroxydans*, an uncultured Sutterellaceae and *Gallionella* (all Gammaproteobacteria) best differentiated upstream sites from the lower stream sections (Figure 7b). Conversely, an uncultured Spirosomaceae and *Haliscomenobacter* (both class Bacteroidia), uncultured Acidimicrobiiaa (Actinobacteria), *Polynucleobacter* (class Gammaproteobacteria) and *Leptothrix* were significantly more abundant in midstream, and particularly downstream communities.

#### 3.3.3 Spatial patterns: bacterioplankton

Proteobacteria were the dominant group also in bacterioplankton, accounting for up to 58% of the total bacterial abundance. Verrucomicrobacteria (23%), Actinobacteria (5%) and Bdellovibrionota (3.5%) were also abundant members of the bacterioplankton (Figure S3c).

Network position was also strongly related to variation in bacterioplankton community composition (PERMANOVA, global test: *F*_2,101_ = 6.72, *p* < .001) (Figure 5c). In pairwise comparisons, upstream and downstream sites exhibited little overlap (*F*_1,62_ = 9.75, *p* < .001) and the difference between midstream and upstream communities was almost equally strong (*F*_1,78_ = 7.35, *p* < .001). The compositional difference was also significant for the midstream–downstream comparison although the pattern was weaker thereafter (*F*_1,62_ = 2.47, *p* < .001). Water temperature, pH, water velocity (all highest in downstream sites) and nitrate concentration (highest in upstream sites) (Table S4) were the strongest environmental correlates of the spatial ordination solution (Figure 5c). Upstream sites harboured substantially more unique taxa, 44% of all taxa in upstream sites being absent from all downstream sections, compared to midstream (20%) or downstream (8%) sites. Numbers of taxa shared between any combination of sections were very low (<11%), being only 3% between the upstream and downstream sections (Figure 6c). ancom‐bc identified 44 genera as differentially abundant between upstream and downstream sites, and 30 between upstream and midstream sites. An uncultured member of Acidobacteria subgroup‐2 and uncultured Nitrospirota class 4‐29‐1 were the most differentially abundant taxa, with higher abundances in upstream sites. *Polynucleobacter*, *Leptothrix*, hgcl‐clade (Actinobacteria), Candidatus *Planktoluna* (Actinobacteria) and Candidatus *Methylopumilus* (Gammaproteobacteria) were all distinctly more abundant in midstream and downstream than in upstream sections (Figure 7c).

#### 3.3.4 Phylogenetical community composition

**FIGURE 8:**
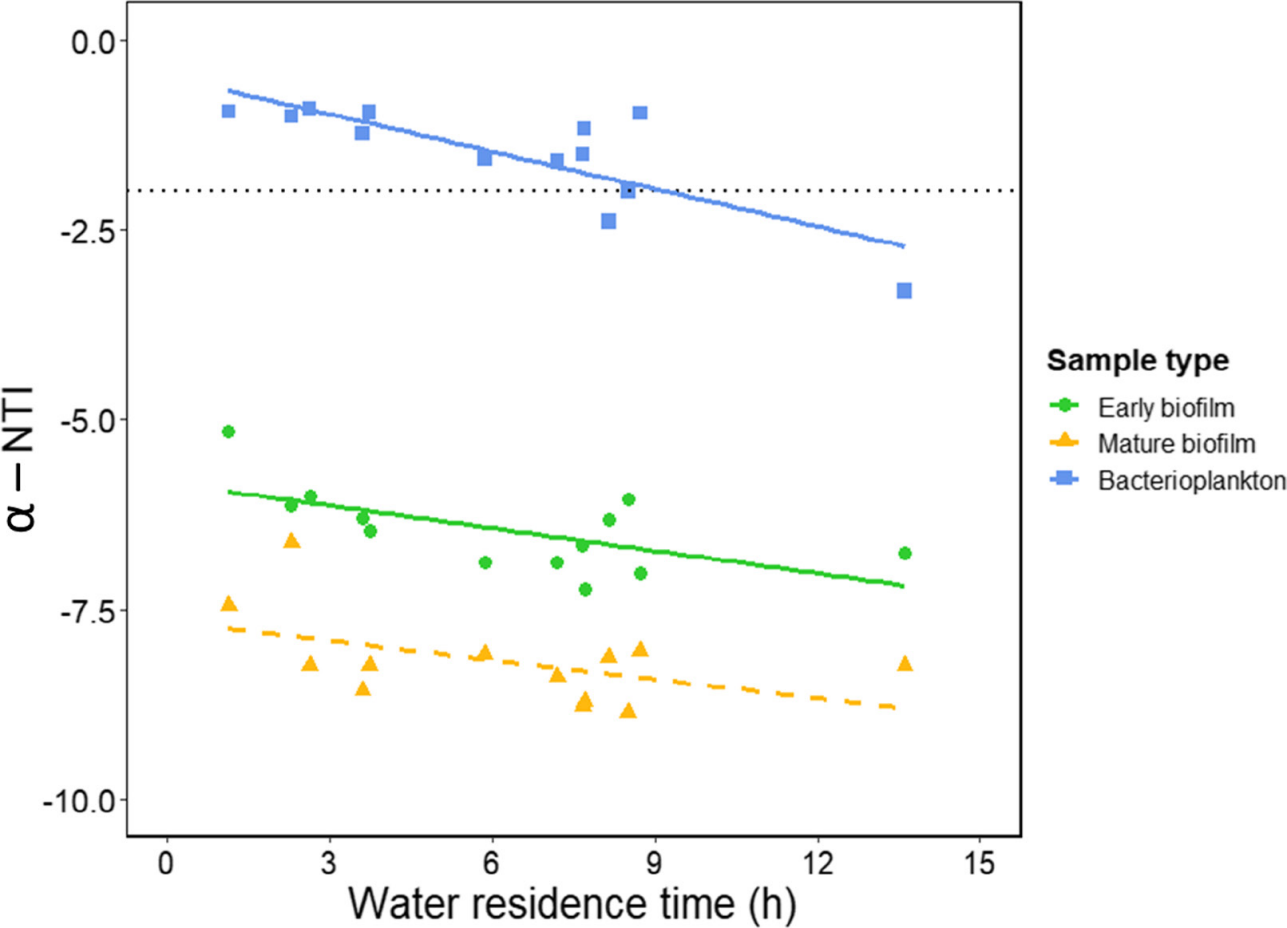
Phylogenetic community structure within communities (α‐NTI) at different water residence times. Data pooled across sampling times. α‐NTI is presented separately for each sample type: Early biofilm, mature biofilm and bacterioplankton. NTI less than −2 indicates phylogenetic clustering (taxa more closely related than expected by chance). Significant patterns are displayed as solid lines.

NTI of bacterioplankton decreased with increasing water residence time (*R* ^2^ = .643, *p* = .001). A similar, albeit weaker, spatial trend was observed for early biofilm (*R* ^2^ = .384, *p* = .024) while mature biofilm showed no spatial pattern (Figure 8). NTI was consistently higher in bacterioplankton than in either of the biofilm communities, indicating generally higher phylogenetic relatedness among biofilm than among bacterioplankton ASVs. Biofilm communities were strongly phylogenetically clustered at all sites in the seasonally pooled data while bacterioplankton did not deviate from randomness until the most downstream site where the community was phylogenetically clustered (Figure 8). Seasonally specified data showed a decreasing trend in NTI, that is more phylogenetically clustered communities towards autumn, for both early and mature biofilm (*F*_2,20_ = 5.12, *p* = .016; *F*_2,20_ = 12.68, *p* < .001, respectively) and stronger phylogenetic clustering for bacterioplankton around midsummer than towards autumn (*F*_2,20_ = 5.75, *p* = .011), especially at downstream sites (Figure S5).

#### 3.3.5 Seasonal community patterns

Early biofilm communities were clearly seasonally structured (PERMANOVA: *F*_2,101_ = 6.24, *p* < .001). Pairwise comparisons showed that the midsummer communities differed distinctly from both early summer (*F*_1,63_ = 6.78, *p* < .001) and autumnal (*F*_1,76_ = 7.52, *p* < .001) communities whereas the latter two differed less (although still significantly) (*F*_1,63_ = 3.68, *p* < 0.001) (Figure 5a). As much as 45% of the midsummer ASVs were unique, while the corresponding numbers were 7% in early summer and 14% in autumn. The strongest environmental correlates of the seasonal community variability were largely the same for both biofilm stages, that is water temperature, pH and, for mature biofilm, nitrate concentration (Figure 5a).

Mature biofilm communities showed much weaker seasonal structuring (*F*_2,88_ = 1.79, *p* = .005), with midsummer communities differing from those in the autumn (*F*_1,76_ = 2.19, *p* < .001) but much less so from the early‐summer communities (*F*_1,50_ = 1.41, *p* = .002) (Figure 5b).

For bacterioplankton, a different pattern emerged: seasonal communities differed significantly (*F*_1,101_ = 3.99, *p* < .001) but, in this case, early and midsummer samples overlapped more (*F*_1,63_ = 2.07, *p* < .001) while autumn samples differed distinctly from both midsummer (*F*_1,76_ = 5.26, *p* < .001) and early summer (*F*_1,63_ = 4.41, *p* < .001) (Figure 5c). Largely the same environmental variables that were significant correlates for biofilm were also important for bacterioplankton, with the exception of groundwater contribution, which only correlated with bacterioplankton community composition (Figure 5c).

## 4 DISCUSSION

Our aim in this study was to examine if the position of a site within the stream network bears an imprint on the diversity and community composition of riverine bacterial metacommunities in a pristine subarctic watershed. Previous studies have largely focused on bacterioplankton communities, which have been reported to exhibit decreased diversity and a shift in community composition along the network, with upstream sites consisting of a diverse mixture of both freshwater and soil‐derived taxa, the latter of which are rapidly filtered out from the aquatic metacommunity (Hassell et al., 2018; Niño‐García et al., 2016a; Read et al., 2015; Ruiz‐González et al., 2015). Whether benthic biofilm communities exhibit corresponding patterns has been much less studied but they might be expected to vary less along the network because they should respond to largely similar habitat filters at all network positions. As expected, bacterioplankton diversity exhibited a strong spatial pattern of decreasing taxonomic diversity from the smallest headwaters to 3rd‐order downstream sections although distinctly so only during summertime low‐flow conditions. In contrast, biofilm diversity did not show any longitudinal variation. Bacterioplankton phylogenetic community composition also changed along the network, and downstream communities were more phylogenetically clustered than those at the upper stream sections. Biofilm communities were generally more phylogenetically clustered than bacterioplankton but with a much weaker (young biofilm) or nonexistent (mature biofilm) spatial pattern. Both biofilm and bacterioplankton community composition differed consistently at different network positions, with 1st‐order sites supporting communities that showed little similarity with 2nd‐order sites, and even less so with 3rd‐order sites. Community composition varied also seasonally, and in particular midsummer biofilm samples supported several taxa that were not observed in other seasons, probably because they appeared in detectable numbers only under favourable environmental conditions, thus displaying temporal variability indicative of “conditionally rare taxa” (see Shade et al., 2014). Again, bacterioplankton showed a different pattern, with autumnal communities differing most from other seasons.

### 4.1 Spatial patterns: biofilm

Bacterial diversity was generally highest in mature biofilm. The difference between biofilm developmental stages was substantial as early biofilm consistently supported the lowest taxonomic diversity. In a colonization experiment in a prairie stream, Veach et al. (2016) observed a very rapid establishment of bacterial communities: operational taxonomic unit (out) richness was high within a few days from the onset of the experiment and increased only slightly thereafter (although Simpson diversity continued to increase). Biofilm colonization dynamics in our subarctic study system were apparently much slower as ASV richness after 14 days was consistently <50% of that on natural stream stones. This discrepancy may partly reflect the different temporal scale of sampling between the two studies, as our sampling missed the very first days of bacterial colonization. However, the phylum‐level composition of early and mature biofilm was very similar, with one major exception: Cyanobacteria were abundant on natural stones but almost completely absent from the short‐term colonization substrates. Cyanobacteria are known to produce allelopathic chemicals (e.g., Dias et al., 2017) and one might therefore have expected them to affect negatively other biofilm community members (see Besemer et al., 2013); this, however, did not seem to be the case because largely the same taxa were dominant on both early and mature biofilm. Furthermore, Cyanobacteria were abundant members of the mature biofilm in all stream sections, which may partly explain the lack of a longitudinal diversity pattern in mature biofilm. The presence of Cyanobacteria in only mature biofilm reflects successional development whereby early stages of biofilm formation were characterized by heterotrophic bacteria and the degree of autotrophy increased with time (see Jackson, 2003; Veach et al., 2016). Finally, while the absence of Cyanobacteria from tiles could merely reflect the different type of substrate used to collect early vs. mature biofilm, data from another (unpublished) experiment show that Cyanobacteria do colonize the tiles but only after a considerable lag period (see Figure S7).

Our finding of higher diversity in mature biofilm than in the overlying water column concurs with Gweon et al. (2021) who also observed consistently higher diversity in benthic biofilms than in bacterioplankton in the tributaries of the River Thames. Biofilm communities in their study also exhibited less among‐site variability than bacterioplankton, which they interpreted to reflect deterministic filtering in benthic environments that are selective for a large but invariable core community, irrespective of differences in water‐column communities. While this observation seemingly contradicts our finding of clear differences in biofilm community composition relative to network location, it may to a large extent reflect differing sampling designs between the two studies, Gweon et al. (2021) being less focused on network position than our spatially explicit design. In our study, biofilm communities were much less (albeit still significantly) longitudinally separated than were bacterioplankton communities, the latter supporting upstream communities that differed distinctly from those at lower network positions.

### 4.2 Spatial patterns: bacterioplankton

Bacterioplankton was less diverse than bacterial communities in mature biofilm at downstream sites, reflecting the strong longitudinal reduction in bacterioplankton diversity. A similar negative spatial trend in bacterioplankton diversity, usually with a drop in diversity at 3rd‐ or 4th‐order sites, has been frequently reported (e.g., Nino‐Garcia et al., 2016b; Ruiz‐González, Niño‐García, Berggren, & del Giorgio, 2017b; Teachey et al., 2019) and has usually been interpreted as the loss of soil‐derived “tourist” taxa with increasing distance from the source (Niño‐García et al., 2016b; Ruiz‐González, Niño‐García, Berggren, & del Giorgio, 2017b). We observed a corresponding loss of diversity in our data already at very short distances (from 1st‐ to 2nd‐order sites) although more distinctly so when dendritic distance approached 4000 m (3rd‐order sites). Of the three Hill numbers, the decrease was sharpest for inverse Simpson index, indicating the strong dominance in downstream sections by a few abundant taxa. In all network positions, taxa that predominated the bacterioplankton community were characteristic freshwater species, and soil‐derived taxa mainly contributed to the diversity peak at the upmost headwater sites. These taxa apparently cross the land–water interface during exceptional rainfall events (Caillon et al., 2021; Cruaud et al., 2020), leading Wisnoski and Lennon (2021) to argue that local‐scale connectivity between terrestrial soils and bacterioplankton is in fact transient and relatively weak (see also Monard et al., 2016). By far most of the soil‐derived taxa are poorly adapted to freshwater life and are therefore rapidly filtered out from the aquatic species pool and/or remain metabolically inactive in the aquatic environment. Indeed, this is exactly what Wisnoski et al. (2020) observed along a hydrological flow path from upstream soils to a reservoir: while the total water‐column community exhibited a sharp decrease in diversity, the active part of it remained unaltered. Although the spatial distance in their study was much shorter than ours, their active community behaved much like the early‐biofilm community in our data, with a similar lack of a shift in diversity with dendritic distance. It may therefore be that the early colonizers of the bare substratum in our study were mainly metabolically active; indeed, it has been suggested that biofilm microbial communities represent a metabolically active component of the stream microbiota (Veach et al., 2016). Consequently, the high bacterioplankton diversity at the upmost headwaters may not translate to a hotspot of ecosystem activity because a large share of this diversity is created by nonaquatic organisms that are passively transported with flowing water and do not become established in metabolically active local communities. There were, however, notable exceptions to this pattern: as predicted by the “core seed bank” hypothesis (Ruiz‐González et al., 2017a), some of the taxa that were abundant in downstream sections were already found in the upmost headwaters, and even in the riparian soils, but where they were mainly very rare (relative abundance <0.01%). These taxa may also represent aquatic bacteria that remained dormant in upstream soils and became active soon after having entered the aquatic ecosystem (see Fazi et al., 2008). Accordingly, our phylogenetical analysis revealed that downstream bacterioplankton consisted of relatively closely related taxa adapted to freshwater life while upstream communities were phylogenetically much more diverse.

### 4.3 Seasonal patterns: local and regional drivers of stream bacterial metacommunities

The key environmental factors related to bacterial community composition were largely the same for both biofilm and bacterioplankton communities, and also for spatial as well as seasonal variation: water temperature and pH. These same variables have been repeatedly reported to be the main correlates of bacterial communities in catchment‐scale (or broader) studies (e.g., Lear et al., 2008; Niño‐García et al., 2016a; Teachey et al., 2019). In our study system, both these variables change systematically with network position, increasing with distance from the stream source. Hydrological variables and stream hydraulics have also been observed to be among the key determinants of both bacterioplankton (Niño‐García, et al. 2016) and biofilm (Niederdorfer et al., 2016; Widder et al., 2014) communities. It is of course possible that we did not measure the most relevant hydrological variables (e.g., near‐bed hydraulics and flow dynamics), or measured them at an inappropriate scale, and therefore we may have underestimated their importance.

For biofilm communities, local habitat variables may indeed represent the key environmental filters but as most bacterioplankton taxa in headwaters are likely to be inactive, any links between community composition and the local environment may be ambiguous (Niño‐García et al., 2016b; Wisnoski et al., 2020). Hassell et al. (2018) detected a consistently high diversity of water‐column bacteria on two of five sampling occasions in a temperate stream network. They suggested that the disruption of the longitudinal diversity pattern on those dates was caused by increased precipitation that resulted in an increased abundance of soil‐associated bacteria in downstream sites. Another factor reducing the importance of network position was linked to unusually low water temperature which caused a shift in the environmental filtering process (Hassell et al., 2018). In a similar vein, Stadler and del Giorgio (2022) documented a seasonal shift in the relative importance of bacterioplankton assembly mechanisms in a boreal watershed, with mass effects dominating in spring and species sorting dominating in summer. This shift was related to seasonal hydrological variation, particularly stream discharge and water residence time, but also water temperature. In our study, bacterioplankton diversity was primarily controlled by water level fluctuation, mainly reflecting seasonal variation in precipitation. Finnish streams and rivers are characterized by a highly predictable discharge regime, with snowmelt‐induced spring flood, minimum flows during summer and a secondary peak in autumn, as a result of increased rainfall and decreased evapotranspiration (Korhonen & Kuusisto, 2010). This discharge regime was also observed in River Riisijoki, resulting in the loss of the longitudinal diversity pattern as the water level rose towards autumn and more taxa of terrestrial origin were transported further down along the network. Thus, much of the seasonal variability in spatial patterns of bacterioplankton may be explained by the interplay between local habitat factors and regional processes whose relative importance varies seasonally.

## 5 CONCLUSIONS

We demonstrated in this study distinct spatial (within‐network) patterns in taxonomic diversity, community composition and phylogenetic relatedness of bacterial communities in a subarctic stream network. These patterns were particularly strong for water‐column communities, whereas both young and mature biofilm exhibited weaker patterns. In particular, we detected (i) higher phylogenetic relatedness within biofilm than bacterioplankton communities, (ii) increased phylogenetic relatedness of bacterioplankton along the hydrological flow path from upstream headwaters to 3rd‐order reaches, and (iii) the dependence of the longitudinal bacterioplankton diversity pattern on hydrological variability. Our results add to the body of evidence showing that water‐column and biofilm communities in stream networks are shaped by different processes, representing largely separate microbial lifestyles (sensu Niederdorfer et al., 2016). Our research thus demonstrates the benefits of studying bacterioplankton and biofilm communities simultaneously to allow for a stronger test of ecological hypotheses about the key patterns in freshwater bacterial assemblages and the mechanisms underlying these patterns.

## AUTHOR CONTRIBUTIONS

TM and KLH conceptualized and designed the study. All authors were involved in the field sampling. Laboratory work and bioinformatics analysis were done by JM with the supervision of KL. Analysis of data was done by JM, KLH, TM, JN and JJ. The manuscript was written by TM, JM and KLH with input from all authors.

## CONFLICT OF INTEREST

The authors have declared no conflict of interest for this article.

## Supporting information


Figure S1‐S7‐Table S1‐S4

## Data Availability

Raw sequence reads of individual samples with their corresponding metadata are deposited in NCBI SRA under BioProject # PRJNA821862. The BioSample file was constructed using MIMARKS Survey version 5.0 package.
