# Supplementary material for: Bacterial communities in a subarctic stream network: Spatial and seasonal patterns of benthic biofilm and bacterioplankton
Source: Mol Ecol. 2022 Oct 17;31(24):6649–63. doi: 10.1111/mec.16711 (PMC10091710; doi:10.1111/mec.16711)
Supplement: Supplementary file 1 — Figure S1‐S7‐Table S1‐S4 [file MEC-31-6649-s001.pdf]

MOLECULAR ECOLOGY

**Supplemental Information for:**

# Bacterial communities in a subarctic stream network: spatial and seasonal patterns of benthic biofilm and bacterioplankton

Jacqueline Malazarte, Timo Muotka, Jussi Jyväsjärvi, Kaisa Lehosmaa, Joel Nyberg & Kaisa-Leena Huttunen

## Table of Contents:


| | |
|---|---|
| Fig. S1. Cumulative precipitation and groundwater contribution | Page 2 |
| Fig. S2. Hill number 1 (Shannon) & 2 (inverse of Simpson) | 3 |
| Fig. S3. Major bacterial phyla | 4 |
| Fig. S4. Major bacterial genera | 5 |
| Fig. S5. Seasonal α-NTI | 6 |
| Fig. S6. Relationship of upstream soil communities to bacterioplankton | 7 |
| Fig. S7. Cyanobacteria abundance (natural stone vs. tile surfaces) | 8 |
| Table S1. Dominant taxa ID – early biofilm | 9 |
| Table S2. Dominant taxa ID – mature biofilm | 10 |
| Table S3. Dominant taxa ID – bacterioplankton | 11 |
| Table S4. Key environmental variables | 12 |

**Fig. S1**. Seven-day cumulative precipitation preceding each sampling date (a) and groundwater contribution (b) across time and network positions.

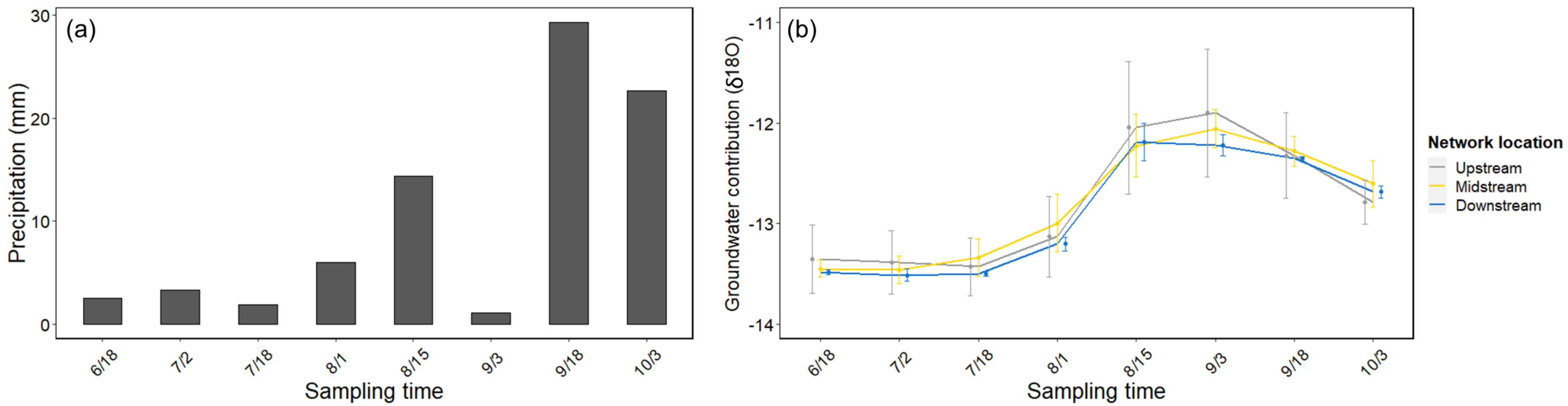

**Fig. S2.** Diversity as Hill number 1 (Shannon entropy) and 2 (inverse of Simpson index) at each of the three network positions (upstream; midstream; downstream) for early biofilm (a, d), mature biofilm (b, e) and bacterioplankton (c, f). Boxes represent median values (horizontal lines) with upper and lower quartiles; whiskers indicate the range of nonoutliers.

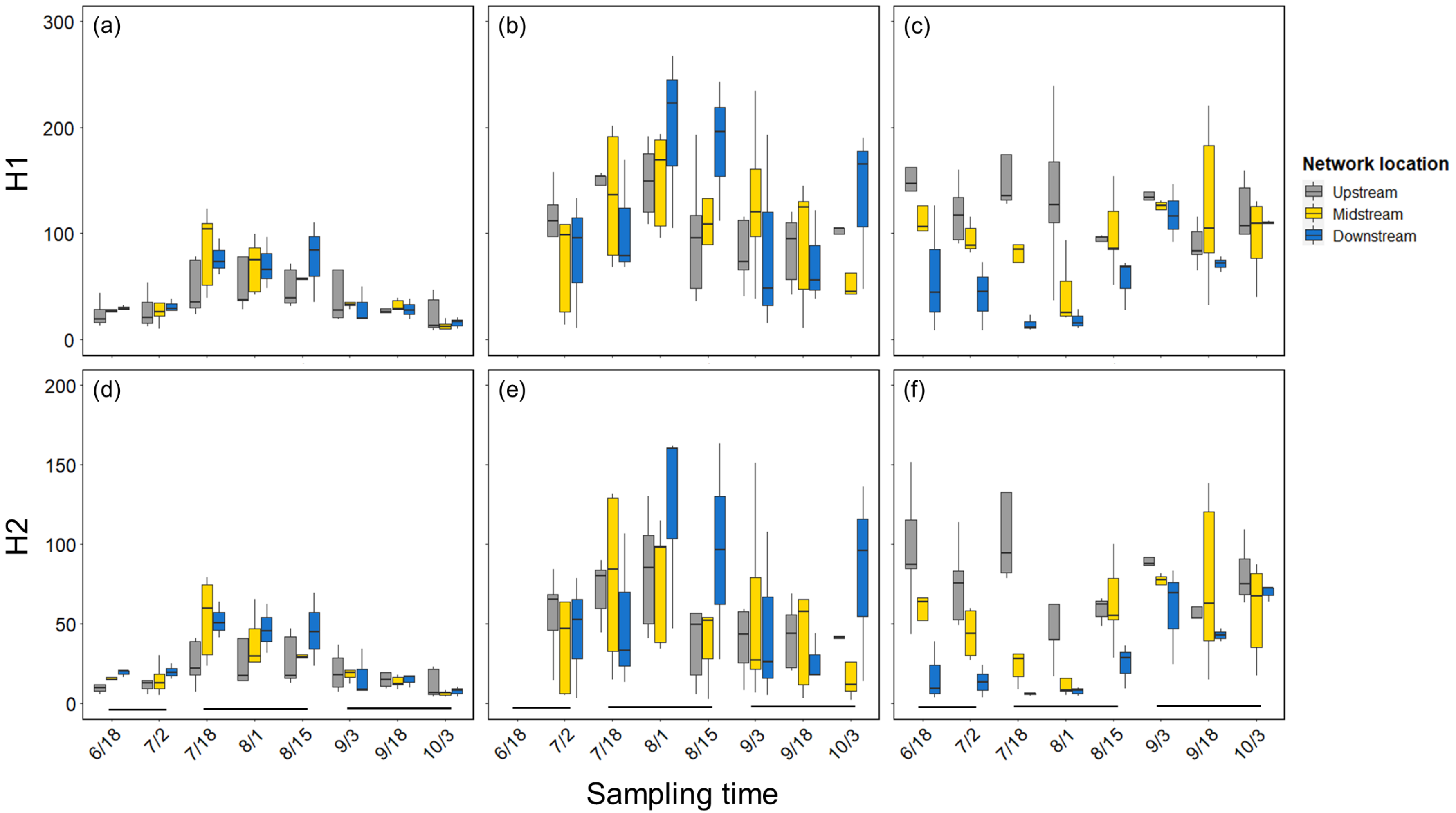

**Fig. S3**. Relative proportions of major bacterial phyla in different network positions for early biofilm (a), mature biofilm (b), and bacterioplankton (c). Data pooled across sampling times.

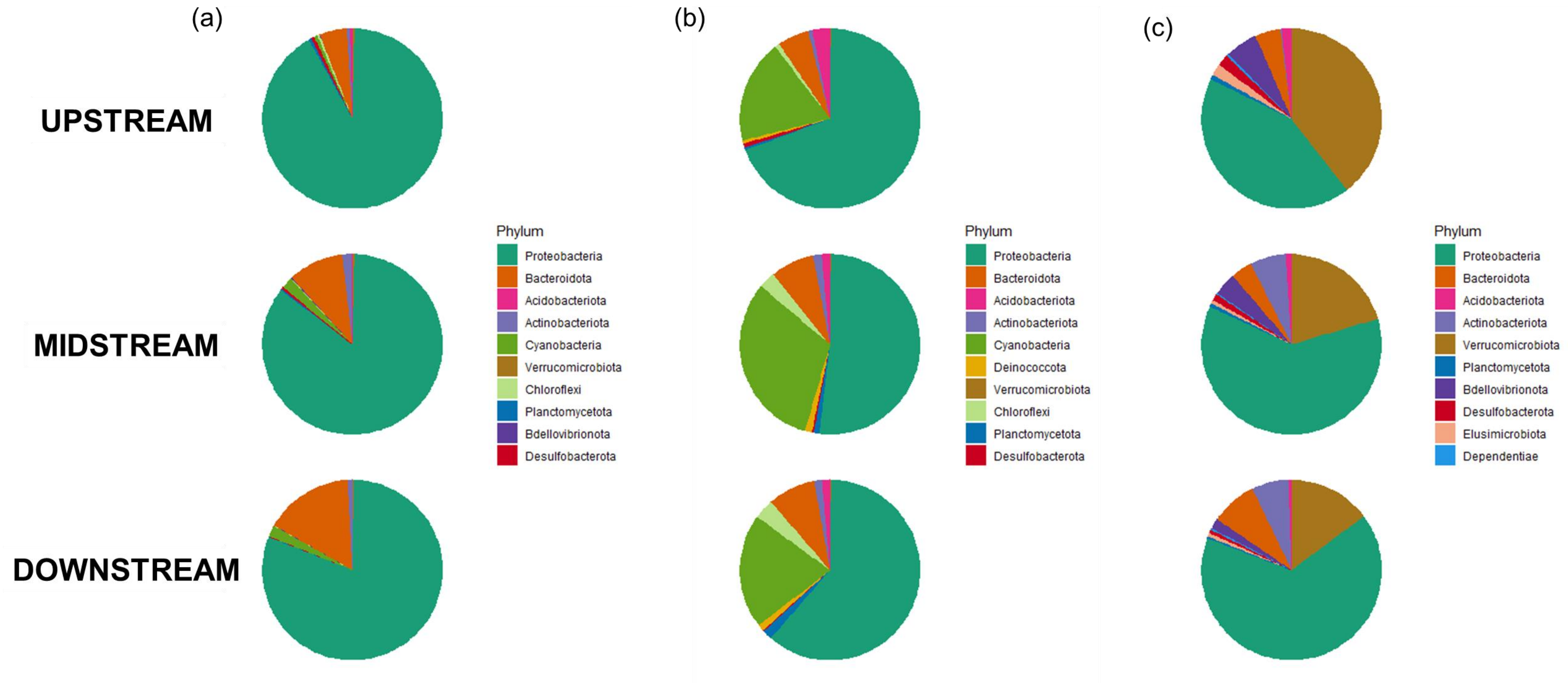

**Fig. S4.** Relative abundances of major bacterial genera at different network positions in early biofilm (a), mature biofilm (b) and bacterioplankton (c). Data pooled across sampling times.

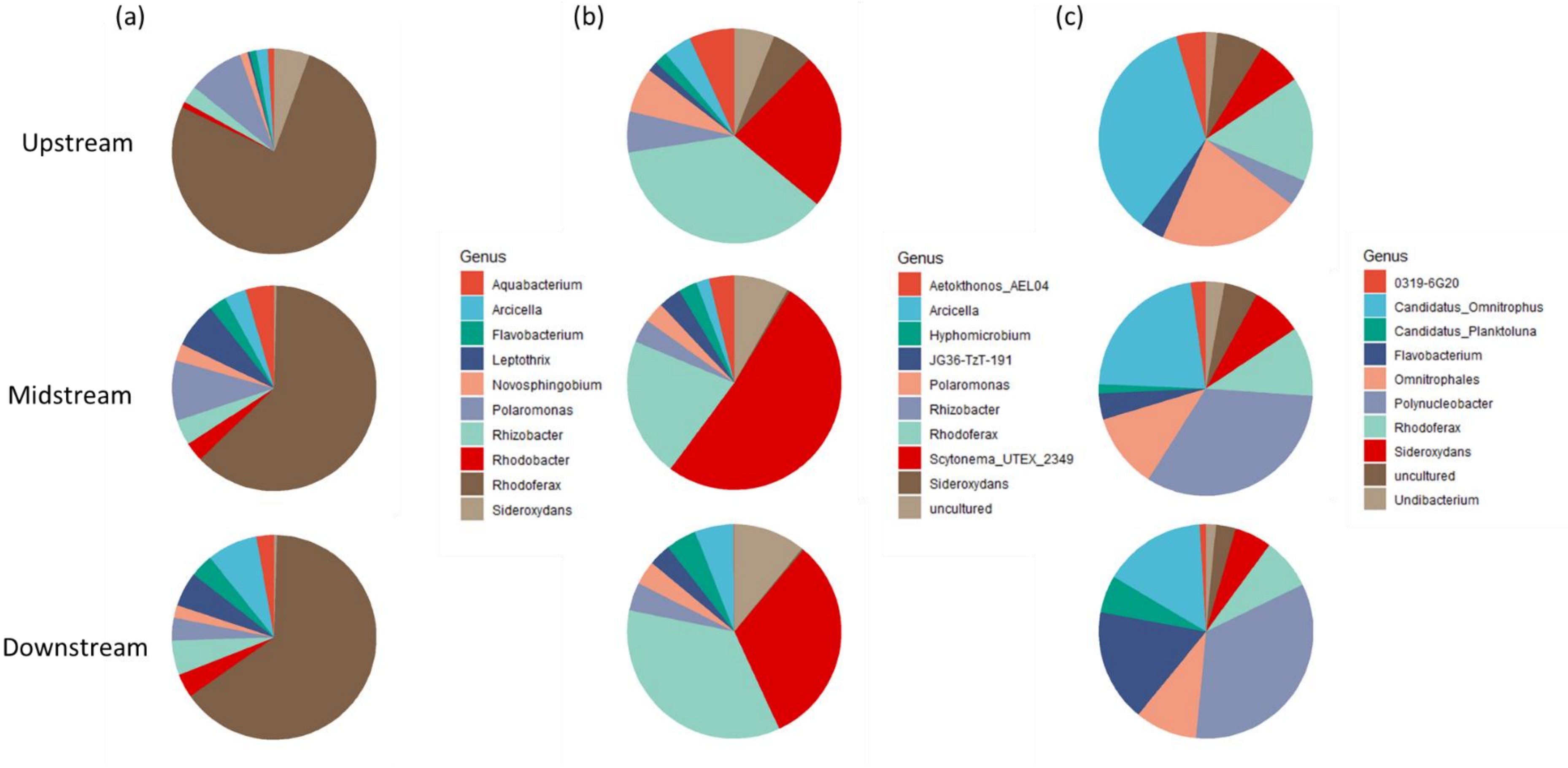

**Fig. S5.** Phylogenetic community structure within communities (α-NTI) at different seasons. Data pooled across sampling sites.

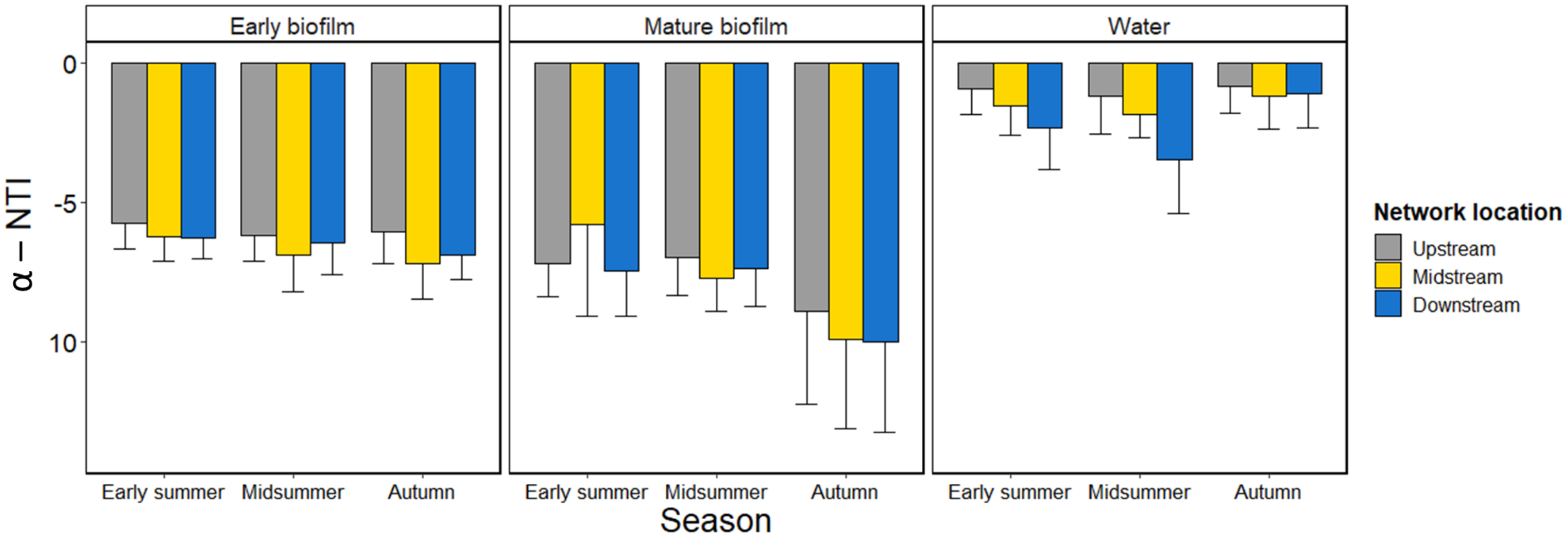

**Fig. S6.** NMDS ordination diagram displaying the relationship of headwater soil communities to bacterioplankton at different network positions. The ellipses represent 95% confidence ellipses around group centroids. For PERMANOVA test results, see text.

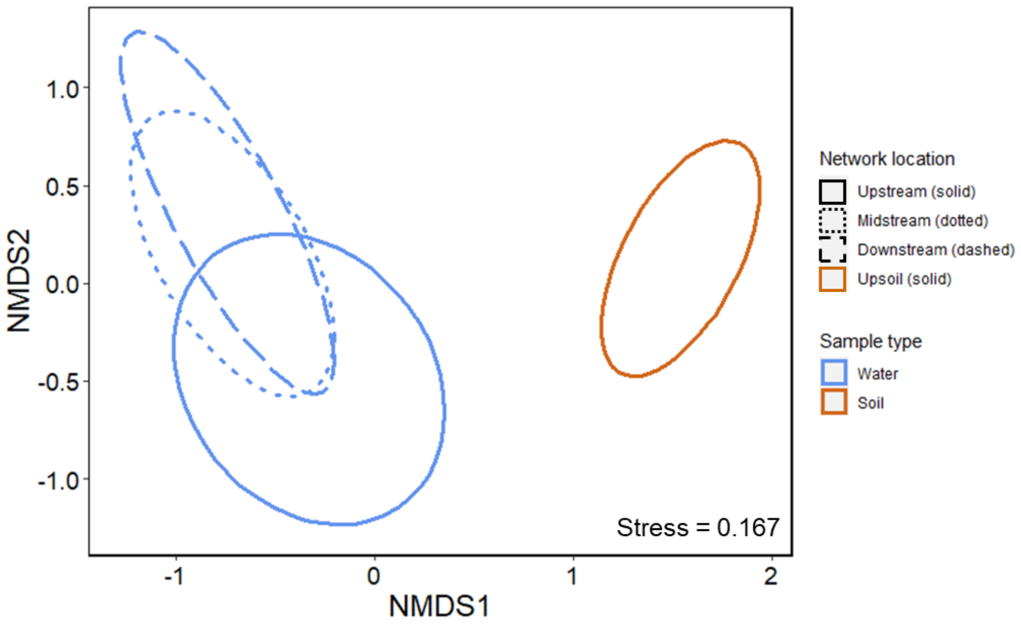

**Fig. S7.** Relative abundance (mean ± 1 SD) of cyanobacteria on natural stones vs. tile surfaces after 4 and 8-week colonization periods.

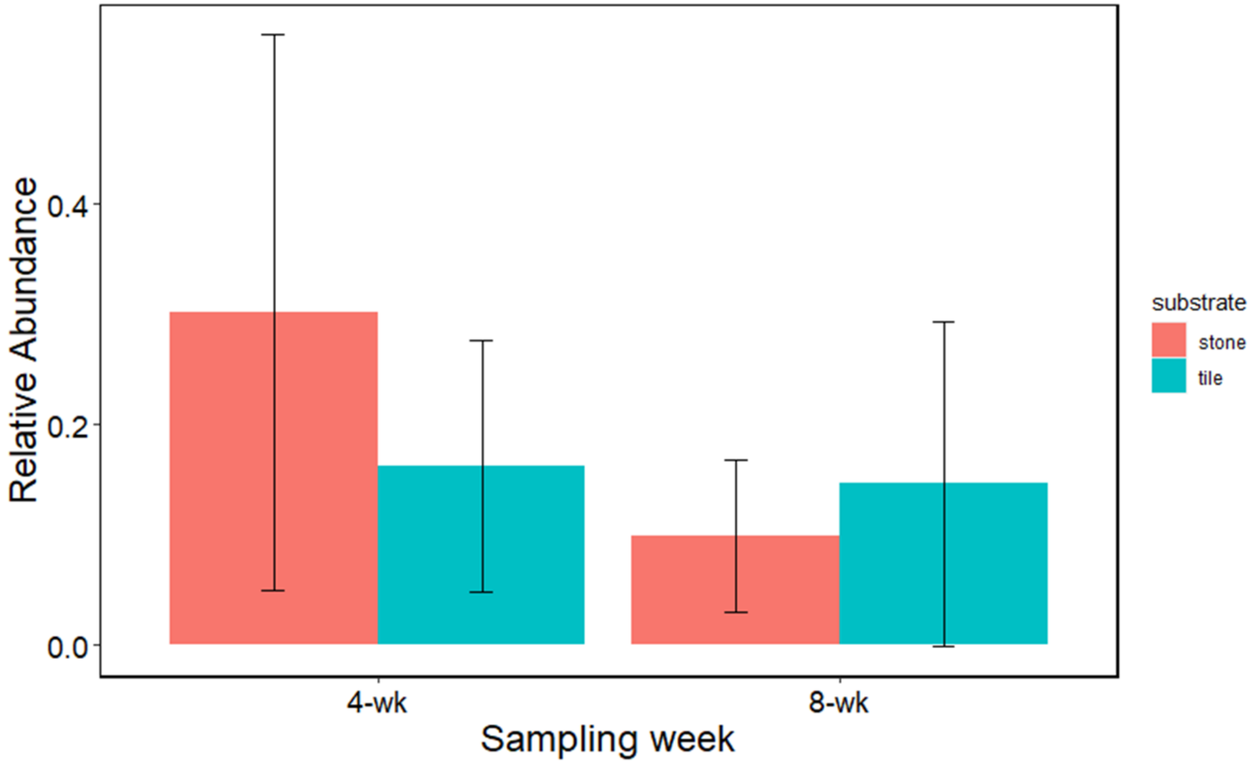

**Table S1.** The five most dominant taxa and their relative abundances in early biofilm communities at each sampling time for upstream, midstream and downstream sites. The most dominant taxa for each sampling time is indicated by grey background. The last column presents the number of times that a certain taxon was the most dominant one. The lowest row presents the cumulative abundance of the five dominant taxa at each sampling time and the last bolded cell is the average cumulative abundance across time.

| Location | Taxon (Phylum;Class;Order;**Family**;Genus;Species) | Jun-18 | Jul-02 | Jul-18 | Aug-01 | Aug-15 | Sep-03 | Sep-18 | Oct-03 | Times rank 1 |
|---|---|---|---|---|---|---|---|---|---|---|
| **Upstream** | NA;NA;NA;**NA**;NA;NA_1 | 4.1 | | | | | | | | *0* |
| | Proteobacteria;Gammaproteobacteria;Burkholderiales;**Comamonadaceae**;NA;NA_1 | 4.8 | 4.2 | 2.6 | 2.7 | 3.1 | | | | *0* |
| | Proteobacteria;Gammaproteobacteria;Burkholderiales;**Comamonadaceae**;NA;NA_2 | 3.7 | 4.9 | 3.3 | 2.6 | | 4.0 | | | *0* |
| | Proteobacteria;Gammaproteobacteria;Burkholderiales;**Comamonadaceae**;NA;NA_3 | | 3.7 | 2.4 | | | | | 5.5 | *0* |
| | Proteobacteria;Gammaproteobacteria;Burkholderiales;**Comamonadaceae**;NA;NA_4 | | | | 2.6 | | | | | *0* |
| | Proteobacteria;Gammaproteobacteria;Burkholderiales;**Comamonadaceae**;NA;NA_5 | | | | | 3.9 | | | | *0* |
| | Proteobacteria;Gammaproteobacteria;Burkholderiales;**Comamonadaceae**;NA;NA_6 | | | | | | | 3.2 | | *0* |
| | Proteobacteria;Gammaproteobacteria;Burkholderiales;**Comamonadaceae**;NA;NA_7 | | | | | | | | 3.3 | *0* |
| | Proteobacteria;Gammaproteobacteria;Burkholderiales;**Comamonadaceae**;Polaromonas;NA | | 4.9 | | | | 5.7 | | 4.6 | *0* |
| | Proteobacteria;Gammaproteobacteria;Burkholderiales;**Comamonadaceae**;Rhodoferax;NA_1 | 27.1 | 24.2 | 15.1 | 12.8 | 13.2 | 17.8 | 14.4 | 25.2 | ***8*** |
| | Proteobacteria;Gammaproteobacteria;Burkholderiales;**Comamonadaceae**;Rhodoferax;NA_2 | 3.7 | | | | 3.1 | 4.7 | 9.2 | 9.3 | *0* |
| | Proteobacteria;Gammaproteobacteria;Burkholderiales;**Comamonadaceae**;Rhodoferax;NA_3 | | | | | | | 3.7 | | *0* |
| | Proteobacteria;Gammaproteobacteria;Burkholderiales;**Gallionellaceae**;NA;NA | | | 2.6 | 3.5 | 4.5 | 4.5 | 3.4 | | *0* |
| | | 43.4 | 41.8 | 26.0 | 24.2 | 27.9 | 36.7 | 33.9 | 47.9 | **35.2** |

| Location | Taxon (Phylum;Class;Order;**Family**;Genus;Species) | Jun-18 | Jul-02 | Jul-18 | Aug-01 | Aug-15 | Sep-03 | Sep-18 | Oct-03 | Times rank 1 |
|---|---|---|---|---|---|---|---|---|---|---|
| **Midstream** | Actinobacteriota;Actinobacteria;Micrococcales;**Microbacteriaceae**;NA;NA | | | 3.3 | | | | | | *0* |
| | Cyanobacteria;Cyanobacteriia;Leptolyngbyales;**Leptolyngbyaceae**;Calothrix_KVSF5;uncultured_bacterium | | | | | 3.6 | | | | *0* |
| | NA;NA;NA;**NA**;NA;NA_1 | 5.3 | | | | | | | | *0* |
| | Proteobacteria;Gammaproteobacteria;Burkholderiales;**Comamonadaceae**;Aquabacterium;NA | | | | | | 3.9 | 4.1 | | *0* |
| | Proteobacteria;Gammaproteobacteria;Burkholderiales;**Comamonadaceae**;Leptothrix;NA | | | 2.7 | 4.5 | 7.4 | | | | *0* |
| | Proteobacteria;Gammaproteobacteria;Burkholderiales;**Comamonadaceae**;NA;NA_1 | | | 2.4 | 2.4 | 4.6 | 3.8 | 5.9 | | *0* |
| | Proteobacteria;Gammaproteobacteria;Burkholderiales;**Comamonadaceae**;NA;NA_2 | 8.1 | 4.9 | | | | | | | *0* |
| | Proteobacteria;Gammaproteobacteria;Burkholderiales;**Comamonadaceae**;NA;NA_3 | 6.1 | 7.8 | 3.0 | | 4.2 | 7.6 | 4.4 | 5.6 | *0* |
| | Proteobacteria;Gammaproteobacteria;Burkholderiales;**Comamonadaceae**;NA;NA_4 | 2.9 | 3.5 | | | | | | | *0* |
| | Proteobacteria;Gammaproteobacteria;Burkholderiales;**Comamonadaceae**;NA;NA_5 | | | | 3.4 | | | | | *0* |
| | Proteobacteria;Gammaproteobacteria;Burkholderiales;**Comamonadaceae**;NA;NA_7 | | | | | | | 5.1 | 6.0 | *0* |
| | Proteobacteria;Gammaproteobacteria;Burkholderiales;**Comamonadaceae**;NA;NA_8 | | | | 3.6 | | | | | *0* |
| | Proteobacteria;Gammaproteobacteria;Burkholderiales;**Comamonadaceae**;Polaromonas;NA | | | | | | 9.6 | | 7.1 | *0* |
| | Proteobacteria;Gammaproteobacteria;Burkholderiales;**Comamonadaceae**;Rhodoferax;NA_1 | 19.1 | 23.6 | 6.2 | 8.1 | 9.0 | 11.0 | 21.4 | 35.4 | ***8*** |
| | Proteobacteria;Gammaproteobacteria;Burkholderiales;**Comamonadaceae**;Rhodoferax;NA_4 | | 2.6 | | | | | | 6.1 | *0* |
| | | 41.5 | 42.4 | 17.4 | 22.1 | 28.9 | 35.9 | 40.9 | 60.2 | **36.2** |

| Location | Taxon (Phylum;Class;Order;**Family**;Genus;Species) | Jun-18 | Jul-02 | Jul-18 | Aug-01 | Aug-15 | Sep-03 | Sep-18 | Oct-03 | Times rank 1 |
|---|---|---|---|---|---|---|---|---|---|---|
| **Downstream** | Bacteroidota;Bacteroidia;Cytophagales;**Spirosomaceae**;Arcicella;uncultured_bacterium | | 5.3 | 3.3 | | | | | 4.8 | *0* |
| | Bacteroidota;Bacteroidia;Flavobacteriales;**Flavobacteriaceae**;Flavobacterium;NA_1 | | | | | 3.3 | | | | *0* |
| | Proteobacteria;Gammaproteobacteria;Burkholderiales;**Comamonadaceae**;Leptothrix;NA | | | 2.4 | 3.9 | 5.2 | | | | ***2*** |
| | Proteobacteria;Gammaproteobacteria;Burkholderiales;**Comamonadaceae**;NA;NA_1 | | | | | 3.1 | 4.2 | 6.0 | 4.7 | *0* |
| | Proteobacteria;Gammaproteobacteria;Burkholderiales;**Comamonadaceae**;NA;NA_10 | | | | 3.4 | | | | | *0* |
| | Proteobacteria;Gammaproteobacteria;Burkholderiales;**Comamonadaceae**;NA;NA_11 | | | | 3.3 | | | | | *0* |
| | Proteobacteria;Gammaproteobacteria;Burkholderiales;**Comamonadaceae**;NA;NA_2 | 6.2 | | | | | | | | *0* |
| | Proteobacteria;Gammaproteobacteria;Burkholderiales;**Comamonadaceae**;NA;NA_3 | 8.4 | 8.6 | | | | 13.9 | 12.0 | 9.9 | *0* |
| | Proteobacteria;Gammaproteobacteria;Burkholderiales;**Comamonadaceae**;NA;NA_4 | | 5.0 | | | | | | | *0* |
| | Proteobacteria;Gammaproteobacteria;Burkholderiales;**Comamonadaceae**;NA;NA_7 | | | | 3.6 | 3.6 | | | | *0* |
| | Proteobacteria;Gammaproteobacteria;Burkholderiales;**Comamonadaceae**;NA;NA_8 | | 4.4 | | | | | | | *0* |
| | Proteobacteria;Gammaproteobacteria;Burkholderiales;**Comamonadaceae**;NA;NA_9 | 4.6 | | 2.8 | | | | | | *0* |
| | Proteobacteria;Gammaproteobacteria;Burkholderiales;**Comamonadaceae**;Rhizobacter;NA_1 | | | | 3.2 | | | | | *0* |
| | Proteobacteria;Gammaproteobacteria;Burkholderiales;**Comamonadaceae**;Rhodoferax;NA_1 | 12.8 | 12.7 | 4.3 | | 4.3 | 14.3 | 17.6 | 32.1 | ***6*** |
| | Proteobacteria;Gammaproteobacteria;Burkholderiales;**Comamonadaceae**;Rhodoferax;NA_2 | 3.6 | | | | | 4.7 | 4.0 | | *0* |
| | Proteobacteria;Gammaproteobacteria;Burkholderiales;**Comamonadaceae**;Rhodoferax;NA_4 | | | | | | 3.7 | 5.3 | 8.9 | *0* |
| | Proteobacteria;NA;NA;**NA**;NA;NA | | | 2.3 | | | | | | *0* |
| | | 35.5 | 36.0 | 15.2 | 17.3 | 19.6 | 40.7 | 44.9 | 60.4 | **33.7** |

**Table S2.** The five most dominant taxa and their relative abundances in mature biofilm communities at each sampling time for upstream, midstream and downstream sites. For other explanations, see Table S1.

| Location | Taxon (Phylum;Class;Order;**Family**;Genus;Species) | Jun-18 | Jul-02 | Jul-18 | Aug-01 | Aug-15 | Sep-03 | Sep-18 | Oct-03 | Times rank 1 |
|---|---|---|---|---|---|---|---|---|---|---|
| **Upstream** | Bacteroidota;Bacteroidia;Cytophagales;**Spirosomaceae**;Arcicella;uncultured_bacterium | | | | | 2.2 | | | | *0* |
| | Cyanobacteria;Cyanobacteriia;Cyanobacteriales;**Nostocaceae**;Aetokthonos_AEL04;Aetokthonos_hydrillicola | | | | 2.4 | | 6.9 | | | ***1*** |
| | Cyanobacteria;Cyanobacteriia;Cyanobacteriales;**Nostocaceae**;Scytonema_UTEX_2349;NA_1 | | 4.0 | | 2.6 | 11.6 | | | 7.2 | ***3*** |
| | Cyanobacteria;Cyanobacteriia;Cyanobacteriales;**Nostocaceae**;Scytonema_UTEX_2349;uncultured_cyanobacterium | | 3.6 | 3.4 | | | | | 4.6 | *0* |
| | Cyanobacteria;Cyanobacteriia;Cyanobacteriales;**Nostocaceae**;Stigonema_SAG_48.90;NA | | | | | 3.8 | | | | *0* |
| | Cyanobacteria;Cyanobacteriia;Cyanobacteriales;**Nostocaceae**;Stigonema_SAG_48.90;Stigonema_sp._1 | | | | | | | | 2.6 | *0* |
| | Cyanobacteria;Cyanobacteriia;Cyanobacteriales;**Nostocaceae**;Stigonema_SAG_48.90;Stigonema_sp._2 | | | | | | | | 1.8 | *0* |
| | Cyanobacteria;Cyanobacteriia;Cyanobacteriales;**Phormidiaceae**;Tychonema_CCAP_1459-11B;NA_1 | | 2.9 | | | | | | | *0* |
| | Proteobacteria;Gammaproteobacteria;Burkholderiales;**Comamonadaceae**;NA;NA_6 | | | 2.3 | | | | 3.9 | | *0* |
| | Proteobacteria;Gammaproteobacteria;Burkholderiales;**Comamonadaceae**;Polaromonas;NA | | | | | | 2.4 | 4.3 | | *0* |
| | Proteobacteria;Gammaproteobacteria;Burkholderiales;**Comamonadaceae**;Rhizobacter;NA_2 | | 2.2 | | 1.3 | | | | | *0* |
| | Proteobacteria;Gammaproteobacteria;Burkholderiales;**Comamonadaceae**;Rhodoferax;beta_proteobacterium | | | | | | | 2.6 | | *0* |
| | Proteobacteria;Gammaproteobacteria;Burkholderiales;**Comamonadaceae**;Rhodoferax;NA_1 | | 3.6 | 3.4 | 2.8 | 2.6 | 5.5 | 5.8 | 3.6 | ***3*** |
| | Proteobacteria;Gammaproteobacteria;Burkholderiales;**Comamonadaceae**;Rhodoferax;NA_2 | | | | | 2.1 | 3.7 | 5.6 | | *0* |
| | Proteobacteria;Gammaproteobacteria;Burkholderiales;**Comamonadaceae**;Rhodoferax;NA_5 | | | 1.5 | 2.2 | | | | | *0* |
| | Proteobacteria;Gammaproteobacteria;Burkholderiales;**Gallionellaceae**;NA;NA | | | | | | 3.5 | | | *0* |
| | Proteobacteria;Gammaproteobacteria;Burkholderiales;**Gallionellaceae**;Sideroxydans;NA | | | 2.1 | | | | | | *0* |
| | | | 16.3 | 12.6 | 11.2 | 22.3 | 22.0 | 22.2 | 19.7 | **18.1** |

| Location | Taxon (Phylum;Class;Order;**Family**;Genus;Species) | Jun-18 | Jul-02 | Jul-18 | Aug-01 | Aug-15 | Sep-03 | Sep-18 | Oct-03 | Times rank 1 |
|---|---|---|---|---|---|---|---|---|---|---|
| **Midstream** | Bacteroidota;Bacteroidia;Cytophagales;**Microscillaceae**;uncultured;metagenome | | | 3.1 | | | | | | *0* |
| | Chloroflexi;Anaerolineae;Caldilineales;**Caldilineaceae**;uncultured;uncultured_Chloroflexia | | | 2.2 | 1.8 | | 2.0 | 4.7 | | *0* |
| | Cyanobacteria;Cyanobacteriia;Cyanobacteriales;**Nostocaceae**;Aetokthonos_AEL04;Aetokthonos_hydrillicola | | | | | | | | 7.1 | *0* |
| | Cyanobacteria;Cyanobacteriia;Cyanobacteriales;**Nostocaceae**;Nostocaceae;Stigonema_tuberculatum | | | | | | | | 3.1 | *0* |
| | Cyanobacteria;Cyanobacteriia;Cyanobacteriales;**Nostocaceae**;Scytonema_UTEX_2349;NA_1 | | 14.1 | 5.6 | 4.8 | 6.4 | 16.1 | 14.3 | 18.7 | ***6*** |
| | Cyanobacteria;Cyanobacteriia;Cyanobacteriales;**Nostocaceae**;Scytonema_UTEX_2349;NA_2 | | 8.1 | | | | | | | *0* |
| | Cyanobacteria;Cyanobacteriia;Cyanobacteriales;**Nostocaceae**;Scytonema_UTEX_2349;uncultured_cyanobacterium | | | | | 14.2 | | | 2.0 | ***1*** |
| | Proteobacteria;Gammaproteobacteria;Burkholderiales;**Comamonadaceae**;Leptothrix;NA | | | | 1.2 | 1.9 | | | | *0* |
| | Proteobacteria;Gammaproteobacteria;Burkholderiales;**Comamonadaceae**;NA;NA_1 | | | | 1.8 | 1.8 | 1.5 | 2.3 | | *0* |
| | Proteobacteria;Gammaproteobacteria;Burkholderiales;**Comamonadaceae**;NA;NA_3 | | 2.1 | 2.2 | | | | | | *0* |
| | Proteobacteria;Gammaproteobacteria;Burkholderiales;**Comamonadaceae**;Rhizobacter;NA_2 | | 1.1 | | | | 1.3 | | | *0* |
| | Proteobacteria;Gammaproteobacteria;Burkholderiales;**Comamonadaceae**;Rhodoferax;NA_1 | | 1.8 | 2.0 | 3.6 | 2.6 | 1.3 | 3.7 | 1.9 | *0* |
| | Proteobacteria;Gammaproteobacteria;Burkholderiales;**Comamonadaceae**;Rhodoferax;NA_4 | | | | | | | 2.3 | | *0* |
| | Proteobacteria;Gammaproteobacteria;Burkholderiales;**Comamonadaceae**;Rhodoferax;NA_5 | | 1.0 | | | | | | | *0* |
| | | | 28.2 | 15.1 | 13.1 | 26.9 | 22.1 | 27.3 | 32.7 | **23.6** |

| Location | Taxon (Phylum;Class;Order;**Family**;Genus;Species) | Jun-18 | Jul-02 | Jul-18 | Aug-01 | Aug-15 | Sep-03 | Sep-18 | Oct-03 | Times rank 1 |
|---|---|---|---|---|---|---|---|---|---|---|
| **Downstream** | Bacteroidota;Bacteroidia;Cytophagales;**Microscillaceae**;uncultured;metagenome | | | 2.4 | 1.2 | | | | | *0* |
| | Bacteroidota;Bacteroidia;Cytophagales;**Spirosomaceae**;Arcicella;uncultured_bacterium | | 3.0 | | | | | | | *0* |
| | Chloroflexi;Anaerolineae;Caldilineales;**Caldilineaceae**;uncultured;uncultured_Chloroflexia | | | | 2.2 | 2.3 | | 4.9 | 2.7 | *0* |
| | Cyanobacteria;Cyanobacteriia;Cyanobacteriales;**Nostocaceae**;Nostocaceae;Cyanomargarita_calcarea | | | | | 1.4 | | | | *0* |
| | Cyanobacteria;Cyanobacteriia;Cyanobacteriales;**Nostocaceae**;Scytonema_UTEX_2349;NA_1 | | 20.7 | | | 5.9 | 8.7 | | 8.4 | ***4*** |
| | Cyanobacteria;Cyanobacteriia;Cyanobacteriales;**Nostocaceae**;Stigonema_SAG_48.90;NA | | | | | 1.9 | | | | *0* |
| | Cyanobacteria;Cyanobacteriia;Cyanobacteriales;**Phormidiaceae**;Tychonema_CCAP_1459-11B;NA_2 | | | 8.5 | | | 11.4 | | | ***1*** |
| | Cyanobacteria;Cyanobacteriia;Leptolyngbyales;**Leptolyngbyaceae**;uncultured;NA | | | 2.8 | | | | | | *0* |
| | Planctomycetota;vadinHA49;vadinHA49;**vadinHA49**;vadinHA49;uncultured_bacterium | | | 2.9 | 3.7 | | | | | ***1*** |
| | Proteobacteria;Gammaproteobacteria;Burkholderiales;**Comamonadaceae**;Leptothrix;NA | | | | | | 2.4 | | | *0* |
| | Proteobacteria;Gammaproteobacteria;Burkholderiales;**Comamonadaceae**;NA;NA_1 | | | | | | | 4.1 | 2.0 | *0* |
| | Proteobacteria;Gammaproteobacteria;Burkholderiales;**Comamonadaceae**;NA;NA_3 | | 2.8 | | | | | 6.6 | | *0* |
| | Proteobacteria;Gammaproteobacteria;Burkholderiales;**Comamonadaceae**;Rhizobacter;NA_2 | | | | 1.3 | 1.9 | | | | *0* |
| | Proteobacteria;Gammaproteobacteria;Burkholderiales;**Comamonadaceae**;Rhodoferax;NA_1 | | 3.1 | 2.3 | 1.8 | | 4.2 | 5.8 | 2.9 | *0* |
| | Proteobacteria;Gammaproteobacteria;Burkholderiales;**Comamonadaceae**;Rhodoferax;NA_3 | | | | | | 2.2 | | | *0* |
| | Proteobacteria;Gammaproteobacteria;Burkholderiales;**Comamonadaceae**;Rhodoferax;NA_4 | | | | | | | 10.8 | 2.7 | ***1*** |
| | Proteobacteria;Gammaproteobacteria;Burkholderiales;**Comamonadaceae**;Thiomonas;uncultured_beta | | 1.9 | | | | | | | *0* |
| | | | 31.5 | 19.0 | 10.1 | 13.4 | 29.0 | 32.3 | 18.6 | **22.0** |

**Table S3**. The five most dominant taxa and their relative abundances in bacterioplankton communities at each sampling time for upstream, midstream and downstream sites. Fort other explanations, see Table S1.

| Location | Taxon (Phylum;Class;Order;**Family**;Genus;Species) | Jun-18 | Jul-02 | Jul-18 | Aug-01 | Aug-15 | Sep-03 | Sep-18 | Oct-03 | Times rank 1 |
|---|---|---|---|---|---|---|---|---|---|---|
| **Upstream** | Bacteroidota;Bacteroidia;Flavobacteriales;**Flavobacteriaceae**;Flavobacterium;Flavobacterium_aquatile | 2.4 | | | | | | | | *0* |
| | Bacteroidota;Bacteroidia;Flavobacteriales;**Flavobacteriaceae**;Flavobacterium;NA_2 | 1.8 | | | | | | | | *0* |
| | Bacteroidota;Bacteroidia;Sphingobacteriales;**Sphingobacteriaceae**;Pedobacter;NA | | | | 1.5 | | | | | *0* |
| | Bdellovibrionota;Oligoflexia;0319-6G20;**0319-6G20**;0319-6G20;uncultured_Syntrophobacteraceae_1 | | | 1.7 | | | | | | *0* |
| | Bdellovibrionota;Oligoflexia;0319-6G20;**0319-6G20**;0319-6G20;uncultured_Syntrophobacteraceae_2 | | | | | | | 2.0 | | *0* |
| | Proteobacteria;Gammaproteobacteria;Burkholderiales;**Comamonadaceae**;NA;NA_6 | 1.7 | | | | | | | | *0* |
| | Proteobacteria;Gammaproteobacteria;Burkholderiales;**Comamonadaceae**;NA;NA_9 | | | 1.6 | 3.2 | 2.0 | | | | *0* |
| | Proteobacteria;Gammaproteobacteria;Burkholderiales;**Comamonadaceae**;Rhodoferax;NA_1 | 3.4 | 3.0 | 2.2 | 5.8 | 2.9 | 1.6 | 2.0 | 2.4 | ***4*** |
| | Proteobacteria;Gammaproteobacteria;Burkholderiales;**Comamonadaceae**;Rhodoferax;NA_3 | | 1.7 | | | | 1.5 | | | *0* |
| | Proteobacteria;Gammaproteobacteria;Burkholderiales;**Comamonadaceae**;Schlegelella;NA | | | | 3.6 | | | 4.3 | | *0* |
| | Proteobacteria;Gammaproteobacteria;Burkholderiales;**Gallionellaceae**;Candidatus_Nitrotoga;NA | | | | | | | | 1.7 | *0* |
| | Proteobacteria;Gammaproteobacteria;Burkholderiales;**Gallionellaceae**;Sideroxydans;NA | | | | | | | | 2.1 | *0* |
| | Proteobacteria;Gammaproteobacteria;Xanthomonadales;**Xanthomonadaceae**;Stenotrophomonas;Stenotrophomonas_rhizophila | | | | 2.3 | | | | | *0* |
| | Verrucomicrobiota;Omnitrophia;Omnitrophales;**Omnitrophaceae**;Candidatus_Omnitrophus;metagenome_1 | | 1.6 | 1.6 | | 2.3 | | | | *0* |
| | Verrucomicrobiota;Omnitrophia;Omnitrophales;**Omnitrophaceae**;Candidatus_Omnitrophus;uncultured_Banisveld | | | | | | 1.4 | | | *0* |
| | Verrucomicrobiota;Omnitrophia;Omnitrophales;**Omnitrophaceae**;Candidatus_Omnitrophus;uncultured_planctomycete | | 1.7 | | | 2.1 | 1.8 | 2.6 | 2.3 | *0* |
| | Verrucomicrobiota;Omnitrophia;Omnitrophales;**Omnitrophales**;Omnitrophales;uncultured_planctomycete | 2.6 | 3.0 | 2.0 | | 4.1 | 3.9 | 4.9 | 4.5 | ***4*** |
| | | 11.8 | 11.0 | 9.2 | 16.5 | 13.4 | 10.2 | 15.7 | 12.9 | **12.6** |

| Location | Taxon (Phylum;Class;Order;**Family**;Genus;Species) | Jun-18 | Jul-02 | Jul-18 | Aug-01 | Aug-15 | Sep-03 | Sep-18 | Oct-03 | Times rank 1 |
|---|---|---|---|---|---|---|---|---|---|---|
| **Midstream** | Actinobacteriota;Actinobacteria;Frankiales;**Sporichthyaceae**;hgcI_clade;uncultured_actinobacterium | | | | | | | | 2.6 | *0* |
| | Bacteroidota;Bacteroidia;Flavobacteriales;**Flavobacteriaceae**;Flavobacterium;NA_3 | | | 2.6 | | | | | | *0* |
| | Bdellovibrionota;Oligoflexia;0319-6G20;**0319-6G20**;0319-6G20;NA | | 2.8 | | | | | | | *0* |
| | Proteobacteria;Gammaproteobacteria;Burkholderiales;**Burkholderiaceae**;Polynucleobacter;NA_1 | 3.9 | 5.4 | 4.6 | 4.9 | 3.5 | 4.4 | 8.0 | 7.1 | ***2*** |
| | Proteobacteria;Gammaproteobacteria;Burkholderiales;**Burkholderiaceae**;Polynucleobacter;NA_2 | | | 2.3 | 4.6 | | | 2.8 | 2.5 | *0* |
| | Proteobacteria;Gammaproteobacteria;Burkholderiales;**Comamonadaceae**;NA;NA_11 | | | | 3.3 | | | | | *0* |
| | Proteobacteria;Gammaproteobacteria;Burkholderiales;**Comamonadaceae**;NA;NA_3 | 2.1 | | | | | | | | *0* |
| | Proteobacteria;Gammaproteobacteria;Burkholderiales;**Comamonadaceae**;NA;NA_9 | 11.0 | 9.6 | 17.4 | 27.9 | 5.1 | 5.1 | | | ***6*** |
| | Proteobacteria;Gammaproteobacteria;Burkholderiales;**Comamonadaceae**;Rhodoferax;NA_1 | 2.3 | 2.2 | 2.0 | 2.7 | 2.9 | 2.3 | 2.2 | 2.6 | *0* |
| | Proteobacteria;Gammaproteobacteria;Burkholderiales;**Gallionellaceae**;Sideroxydans;NA | | | | | 2.5 | 2.6 | | | *0* |
| | Verrucomicrobiota;Omnitrophia;Omnitrophales;**Omnitrophaceae**;Candidatus_Omnitrophus;metagenome_2 | 1.9 | 2.2 | | | | | | | *0* |
| | Verrucomicrobiota;Omnitrophia;Omnitrophales;**Omnitrophaceae**;Candidatus_Omnitrophus;uncultured_planctomycete | | | | | | | 2.3 | | *0* |
| | Verrucomicrobiota;Omnitrophia;Omnitrophales;**Omnitrophales**;Omnitrophales;uncultured_planctomycete | | | | | 2.3 | 2.9 | 2.4 | 2.9 | *0* |
| | | 21.2 | 22.2 | 28.9 | 43.5 | 16.3 | 17.3 | 17.6 | 17.8 | **23.1** |

| Location | Taxon (Phylum;Class;Order;**Family**;Genus;Species) | Jun-18 | Jul-02 | Jul-18 | Aug-01 | Aug-15 | Sep-03 | Sep-18 | Oct-03 | Times rank 1 |
|---|---|---|---|---|---|---|---|---|---|---|
| **Downstream** | Actinobacteriota;Actinobacteria;Micrococcales;**Microbacteriaceae**;Candidatus_Flaviluna;uncultured_actinobacterium | | | | 4.0 | | | | | *0* |
| | Actinobacteriota;Actinobacteria;Micrococcales;**Microbacteriaceae**;Candidatus_Planktoluna;NA | | 3.3 | 3.2 | | | | | | *0* |
| | Bacteroidota;Bacteroidia;Flavobacteriales;**Flavobacteriaceae**;Flavobacterium;NA_3 | | | 22.8 | 8.3 | | | | | *0* |
| | Proteobacteria;Gammaproteobacteria;Burkholderiales;**Burkholderiaceae**;Polynucleobacter;NA_1 | 4.7 | 5.2 | 7.5 | 7.0 | 3.6 | 4.1 | 8.3 | 5.2 | ***2*** |
| | Proteobacteria;Gammaproteobacteria;Burkholderiales;**Burkholderiaceae**;Polynucleobacter;NA_2 | 3.5 | 2.6 | 3.6 | 8.5 | 4.7 | 2.4 | | | *0* |
| | Proteobacteria;Gammaproteobacteria;Burkholderiales;**Comamonadaceae**;NA;NA_3 | 2.3 | 2.0 | | | | | 2.7 | | *0* |
| | Proteobacteria;Gammaproteobacteria;Burkholderiales;**Comamonadaceae**;NA;NA_9 | 33.3 | 32.3 | 29.1 | 31.8 | 18.7 | 9.8 | | | ***8*** |
| | Proteobacteria;Gammaproteobacteria;Burkholderiales;**Comamonadaceae**;Rhodoferax;NA_1 | 2.3 | | | | | | 3.2 | 3.0 | *0* |
| | Proteobacteria;Gammaproteobacteria;Burkholderiales;**Gallionellaceae**;Sideroxydans;NA | | | | | 2.7 | 1.7 | 3.4 | 2.2 | *0* |
| | Verrucomicrobiota;Omnitrophia;Omnitrophales;**Omnitrophaceae**;Candidatus_Omnitrophus;metagenome_1 | | | | | | | | 1.8 | *0* |
| | Verrucomicrobiota;Omnitrophia;Omnitrophales;**Omnitrophales**;Omnitrophales;uncultured_planctomycete | | | | | 3.3 | 2.6 | 4.0 | 3.1 | *0* |
| | | 46.1 | 45.4 | 66.3 | 59.6 | 33.1 | 20.6 | 21.5 | 15.3 | **38.5** |

**Table S4.** Mean values (and ranges) for the key environmental variables at different network locations.

| Environmental variable | Upstream | | | Midstream | | | Downstream | | |
|---|---|---|---|---|---|---|---|---|---|
| | Mean | Min | Max | Mean | Min | Max | Mean | Min | Max |
| Water temperature (ºC) | 9.53 | 1.9 | 16.8 | 11.33 | 2.1 | 19.4 | 11.11 | 3.0 | 18.5 |
| Water depth (cm) | 37.9 | 14 | 72 | 26.9 | 10 | 46 | 53.4 | 10 | 134 |
| Water velocity (cm $s^{-1}$) | 11.0 | 1 | 31 | 17.6 | 8 | 31 | 21.8 | 14 | 32 |
| pH | 6.56 | 5.7 | 7.1 | 6.78 | 6.1 | 7.4 | 6.84 | 6.4 | 7.3 |
| Total phosphorus (µg $L^{-1}$) | 9.24 | 3.4 | 25.3 | 11.54 | 0.2 | 23.9 | 9.54 | 4.9 | 18.2 |
| Nitrate + Nitrite (µg $L^{-1}$) | 6.27 | 1.1 | 13.3 | 5.19 | 1.0 | 13.6 | 4.83 | 1.1 | 9.9 |
| DOC (mg $L^{-1}$) | 14.1 | 5.6 | 43 | 11.9 | 5.0 | 27 | 12.5 | 4.8 | 50 |
| Groundwater contribution ($\delta^{18}O$) | -12.79 | -13.90 | -11.04 | -12.80 | -13.69 | -11.72 | -12.89 | -13.56 | -12.04 |
| Chlorophyll-a (µg $cm^{-2}$ per $d^{-1}$) | 0.034 | 0.010 | 0.153 | 0.024 | 0.010 | 0.091 | 0.021 | 0.010 | 0.160 |
| $Absorbance_{436}$ | 0.047 | 0.014 | 0.159 | 0.040 | 0.020 | 0.077 | 0.038 | 0.020 | 0.070 |
